# Blocking IL-10 receptor signaling ameliorates *Mycobacterium tuberculosis* infection during influenza-induced exacerbation

**DOI:** 10.1172/jci.insight.126533

**Published:** 2019-05-16

**Authors:** Sarah Ring, Lars Eggers, Jochen Behrends, Adam Wutkowski, Dominik Schwudke, Andrea Kröger, Alexandra Maximiliane Hierweger, Christoph Hölscher, Gülsah Gabriel, Bianca E. Schneider

**Affiliations:** 1 Junior Research Group Coinfection, Priority Research Area Infections, Research Center Borstel, Leibniz Lung Center, Borstel, Germany.; 2 Core Facility Fluorescence Cytometry, Research Center Borstel, Leibniz Lung Center, Borstel, Germany.; 3 Bioanalytical Chemistry, Priority Research Area Infections, Research Center Borstel, Leibniz Lung Center, Borstel, Germany.; 4 Institute of Medical Microbiology and Hospital Hygiene, Otto-von-Guericke-University Magdeburg, and Innate Immunity and Infection, Helmholtz Centre for Infection Research, Braunschweig, Germany.; 5 Department of Obstetrics and Fetal Medicine, Laboratory for Experimental Feto-Maternal Medicine, University Medical Center Hamburg-Eppendorf, Hamburg, Germany.; 6 Institute for Immunology, Center for Diagnostics, University Medical Center Hamburg-Eppendorf, Hamburg, Germany.; 7 Infection Immunology, Priority Research Area Infections, Research Center Borstel, Leibniz Lung Center, Borstel, Germany.; 8 Research Department Viral Zoonoses - One Health, Heinrich Pette Institute, Leibniz Institute for Experimental Virology, Hamburg, Germany.; 9 Institute of Virology, University of Veterinary Medicine, Hannover, Germany.

**Keywords:** Immunology, Infectious disease, Influenza, Mouse models, Tuberculosis

## Abstract

Epidemiological findings indicate that coinfection with influenza viruses is associated with an increased risk of death in patients suffering from tuberculosis, but the underlying pathomechanisms are not well understood. In this study, we demonstrate that influenza A virus (IAV) coinfection rapidly impairs control of *Mycobacterium tuberculosis* (*Mtb*) in C57BL/6 mice. IAV coinfection was associated with significantly increased bacterial loads, reduced survival, and a substantial modulation of innate and adaptive immune defenses including an impaired onset and development of *Mtb*-specific CD4^+^ T cell responses and the accumulation of macrophages with increased arginase-1 production in the lungs. Our findings strongly indicate that IAV coinfection compromises the host’s ability to control *Mtb* infection via the production of IL-10, which was rapidly induced upon viral infection. The blockade of IL-10 receptor signaling reduced the bacterial load in coinfected mice to a level comparable to that in *Mtb*-only-infected animals. Taken together, our data suggest that IL-10 signaling constitutes a major pathway that enhances susceptibility to *Mtb* during concurrent IAV infection.

## Abstract 2

IL-10R signaling constitutes a major pathway that impairs control of *Mycobacterium tuberculosis* during influenza co-infection.

## Introduction

Tuberculosis (Tb) is the most prevalent bacterial infectious disease in humans and continues to be a major cause of morbidity and mortality worldwide (1). The causative agent, *Mycobacterium tuberculosis* (*Mtb*), is carried by an estimated 2 to 3 billion people globally. A relatively small proportion (5%–10%) of infected people will develop active disease during their lifetime. However, the immune system fails to achieve sterile eradication of the tubercle bacillus. The enormous reservoir of patients with latent Tb constantly leads to new active Tb cases and disease transmission, thus perpetuating the epidemic (2).

The consequences of acute respiratory viral infections in individuals infected with *Mtb* have not been well studied to date. Influenza virus infections lead to substantial morbidity and mortality every year. It is estimated that annual epidemics result in about 3 to 5 million cases of severe illness and about 290,000 to 650,000 deaths (3). In 2009, the WHO reported a substantial number of deaths in patients with chronic respiratory conditions and raised concerns about the possible impact of influenza on patients with active Tb (4). Recent data from South Africa, one of the 22 Tb high-burden countries (estimated incidence of 322,000 active Tb cases in 2017; ref. 1) indicate Tb to be associated with fatal pandemic influenza infections, as 10% of patients with H1N1 who died during the 2009 influenza pandemic in South Africa also had active Tb (5). Moreover, Walaza and colleagues reported influenza virus infection to be associated with increased risk of death among patients hospitalized with pulmonary Tb in South Africa and suggested researching the potential beneficial impact of influenza vaccination among persons with laboratory-confirmed Tb (6). Likewise, during the most devastating pandemic known in human history (the Spanish influenza from 1918 to 1919), patients with Tb were more likely to die from influenza compared with those without Tb (7–10). Together, these epidemiological findings point to an association between Tb-influenza coinfection and increased mortality.

Despite the fact that the first epidemiological studies and case reports date back 100 years, in-depth understanding of the pathomechanisms leading to an aggravated disease progression during Tb-influenza coinfection is lacking. Redford and colleagues demonstrated that pre-exposure to influenza A virus (IAV) impaired long-term control of *Mtb* in mice, which was associated with a significant decrease in survival of coinfected animals compared with those infected with *Mtb* alone (11). Moreover, they showed that IAV-induced type I IFN signaling was associated with impaired control of *Mtb* because bacterial loads in WT but not in IFN-αβ receptor–knockout (*Ifnar1*-knockout) mice were significantly increased 4 weeks after *Mtb* infection. However, the mechanisms by which influenza virus-induced type I IFNs exacerbate *Mtb* infection are not well understood.

There is growing evidence in both humans and mice that type I IFNs are detrimental during infection with *Mtb*. Increased survival of Ifnar1-deficient mice together with the observation that induction of type I IFNs exacerbates disease and bacterial growth demonstrate that type I IFNs promote *Mtb* infection (12–15). In accordance with those results, patients with active Tb displayed a type I IFN–inducible blood transcriptional signature that was resolved upon successful treatment (16). Type I IFNs negatively regulate antibacterial immune responses via multiple mechanisms. For example, they can downregulate macrophage IFN-γ responsiveness, which results in impaired killing of *Mtb* and reduced production of host-protective cytokines such as IL-1β (17). It was recently proposed that type I IFNs and IL-1 cytokine pathways (the latter is indispensable for controlling *Mtb* infection) (18–21) represent distinct and specialized classes of innate inflammatory responses that antagonize each other (22, 23). Moreover, type I IFNs can induce IL-10, an immunosuppressive cytokine that can dampen Th1 responses to *Mtb* infection and impair bacterial clearance (24).

In this study, we conducted coinfection experiments in C57BL/6 mice and challenged *Mtb-*infected mice with an IAV strain isolated during the 2009 pandemic (A/HH/05/09 pH1N1; ref. 25). We show that IAV coinfection substantially modulated anti-*Mtb* immunity with detrimental consequences for the coinfected host and provide evidence that IL-10/IL-10 receptor signaling constitutes a major pathway that enhances susceptibility to *Mtb* during concurrent IAV infection.

## Results

### IAV coinfection impairs control of airborne Mtb infection.

**Figure 1:**
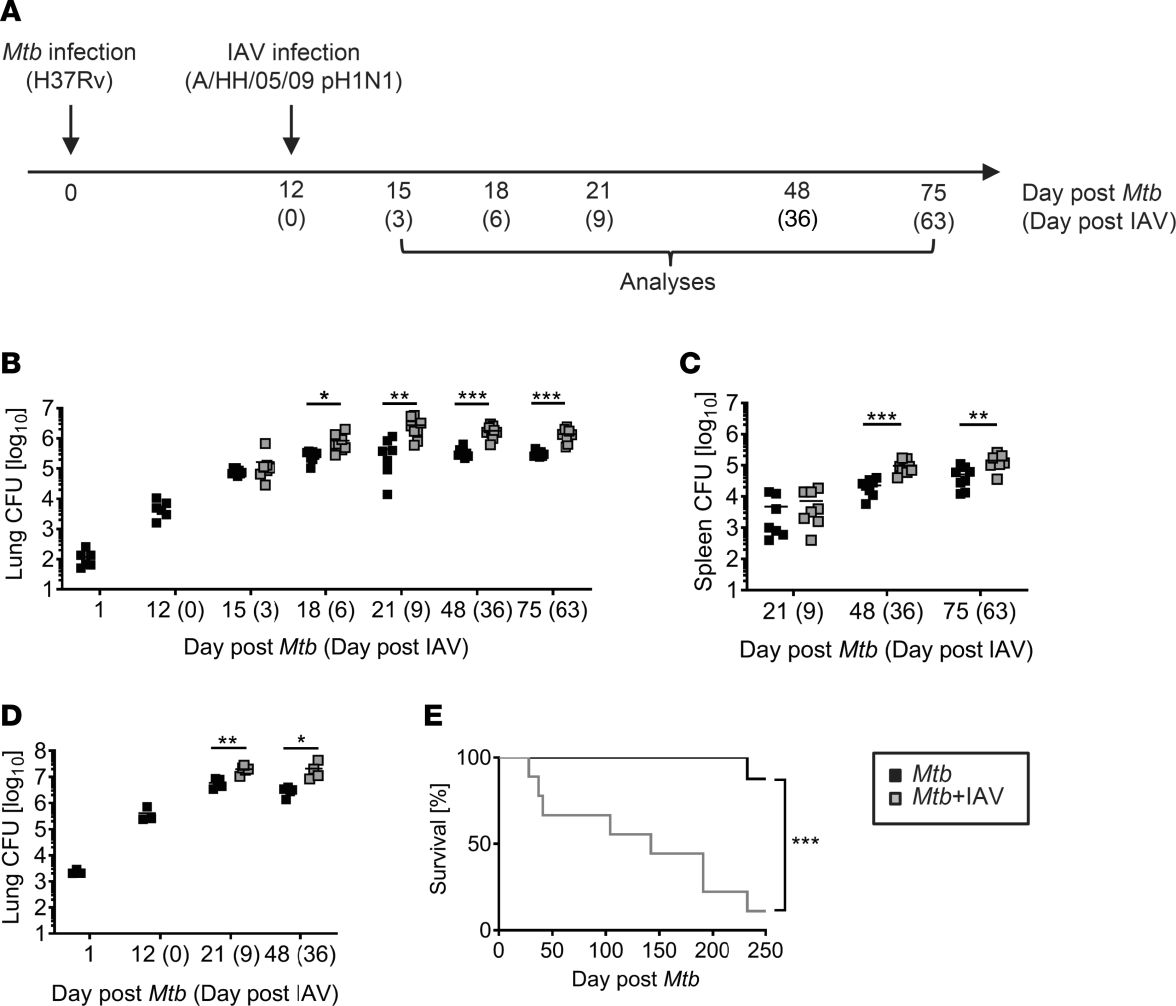
IAV coinfection impairs control of *Mtb*. (**A**) Timeline of experimental setup. (**B** and **C**) C57BL/6 mice were infected via aerosol with a low dose of *Mtb* H37Rv and 12 days later coinfected i.n. with 1 × 10^4^ PFU IAV (A/HH/05/09 H1N1). At indicated time points, bacterial burden in lung (**B**) and spleen (**C**) were determined (*n* = 6 [day 1, day 12 *Mtb*], *n* = 7–9 per group, data pooled from 2 independent experiments). (**D** and **E**) C57BL/6 mice received high-dose *Mtb* H37Rv aerosol infection and were coinfected i.n. with 1 × 10^4^ PFU IAV (A/HH/05/09 H1N1) 12 days later. (**D**) At indicated time points, pulmonary bacterial burden (*n* = 3; day 1, day 12 *Mtb*; *n* = 4–5 per group, data from 1 experiment) was determined. (**E**) Mice were monitored for survival (*n* = 8–9 per group, data from 1 experiment) until day 250 after *Mtb* infection. (**B**–**D**) Each data point represents 1 mouse. *P ≤ 0.05; **P ≤ 0.01, ***P ≤ 0.001 determined by (**B** and **C**) unpaired *t* test and (**E**) log-rank (Mantel-Cox) test.

**Figure 2:**
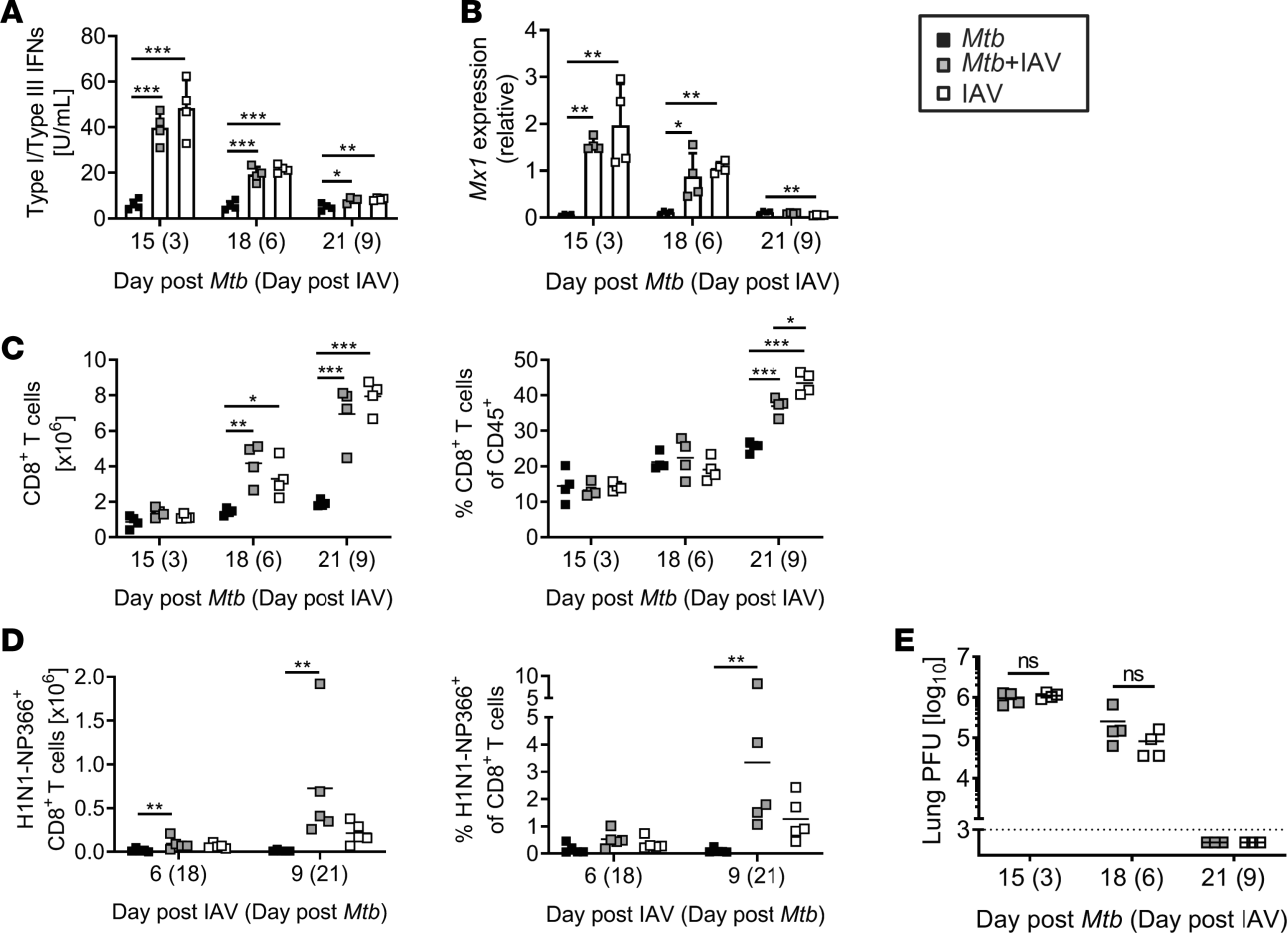
Induction of antiviral immune responses and viral clearance are not impaired in the presence of *Mtb*. C57BL/6 mice were infected via aerosol with a low dose of *Mtb* H37Rv and 12 days later coinfected i.n. with 1 × 10^4^ PFU IAV (A/HH/05/09 H1N1). Lungs collected at indicated time points were analyzed for (**A**) the activity of type I/III IFN and (**B**) *Mx1* expression by qRT-PCR relative to *Gapdh* expression (*n* = 4 per group, representative of 2 independent experiments). (**C**) Shows total lung CD8^+^ T cell numbers and frequencies among CD45^+^ cells (*n* = 4 per group, representative of 2 independent experiments) and (**D**) H1N1-NP366 dextramer–specific CD8^+^ T cells (*n* = 5 per group, 1 experiment). (**E**) Viral titers were determined by MDCK plaque assay (*n* = 3–4 per group, representative of 4 (day 3 and day 6) and 2 (day 9) independent experiments). (**A** and **B**) Data are represented as mean + SD or (**C**–**E**) each point representing 1 mouse. *P ≤ 0.05; **P ≤ 0.01, ***P ≤ 0.001 determined by (**A**–**D**) 1-way ANOVA followed by Tukey’s multiple-comparison test and (**E**) unpaired *t* test.

To experimentally model *Mtb* and IAV coinfection, we infected C57BL/6 mice with low doses of *Mtb* H37Rv via the aerosol route. Twelve days later, mice were challenged with a sublethal dose of IAV (A/HH/05/09 pH1N1) i.n., and Tb disease progression in the presence and absence of the virus was monitored (Figure 1A). As early as 6 days after IAV coinfection (day 18 after *Mtb* infection), the *Mtb* load in the lungs was significantly increased and another 3 days later (day 21 after *Mtb* infection), the *Mtb* burden was approximately 1 log higher when compared with the animals infected with *Mtb* alone (Figure 1B). Importantly, the *Mtb* burden was not only elevated transiently during acute IAV coinfection but also at later time points when IAV infection was resolved (Figure 1B and Figure 2E), indicating that transient IAV coinfection induces sustained impairment of bacterial control. Accordingly, IAV challenge impaired control of high-dose *Mtb* infection, and coinfected mice succumbed significantly earlier than *Mtb-*alone mice (Figure 1, D and E). Together, these data demonstrate the detrimental influence of IAV coinfection during *Mtb* infection. This finding was independent of the time point of coinfection because challenge of mice with IAV 30 days after *Mtb* infection also caused a significant increase in lung mycobacterial levels compared with loads in *Mtb*-alone mice (Supplemental Figure 1; supplemental material available online with this article; https://doi.org/10.1172/jci.insight.126533DS1). In contrast to the lung, which is the primary site of bacterial and viral coinfection, *Mtb* loads in the spleen initially did not differ between single- and coinfected mice, indicating that mycobacterial dissemination to distant organs was not altered by IAV (Figure 1C). However, *Mtb* numbers in the spleen increased significantly at later time points (Figure 1C), indicating impaired control of bacterial replication in the consequence of viral coinfection even at distal sites. In summary, IAV coinfection had an immediate and sustained negative impact on the containment and outcome of aerogenic *Mtb* infection.

### The presence of Mtb does not alter the induction of the antiviral state and viral clearance.

Type I and III IFNs are indispensable mediators of the antiviral defense and are rapidly induced upon influenza virus infection (26, 27). To assess whether the IAV-induced IFN response was altered by a pre-existing *Mtb* infection, we determined IFN I/III activity in whole lung lysates on days 3, 6, and 9 after IAV infection of previously naive or *Mtb-*infected mice. On day 3, IFN I/III activity was significantly higher in the lungs of all IAV-infected animals compared with those infected with *Mtb* alone (Figure 2A). This higher IFN activity in IAV-(co)infected animals rapidly decreased and reached a level similar to that in *Mtb*-only-infected mice after 9 days. The expression of the IFN-stimulated gene *Mx1* in lungs of IAV-(co)infected mice followed a similar pattern of kinetics. After 3 days, *Mx1* expression was significantly increased in IAV-(co)infected lungs compared with those from *Mtb*-only-infected animals but decreased over time and reached comparable low levels in IAV-(co)infected and *Mtb-*infected lungs after 9 days (Figure 2B). Importantly, type I/III IFN activity did not differ in the presence or absence of *Mtb*. For viral clearance, the presence of CD8^+^ T cells is indispensable (28). Total numbers of lung CD8^+^ T cells were significantly increased 6 days after IAV (co)infection, resulting in a higher proportion of CD8^+^ T cells among CD45^+^ cells in IAV-(co)infected compared with *Mtb*-infected lungs (Figure 2C). The number and proportion of CD8^+^ T cells in coinfected mice was comparable to those in IAV-alone mice, indicating that an underlying *Mtb* infection did not compromise the induction of IAV-specific CD8^+^ T cells. In line with this finding, we could detect H1N1 NP366-specific CD8^+^ T cells in IAV-infected mice independent of the presence or absence of *Mtb* (Figure 2D). With the final observation that viral titers did not differ between IAV-only and *Mtb-*IAV-coinfected mice (Figure 2E), we conclude that the antiviral response and viral control were not affected by a pre-existing *Mtb* infection. This finding indicates that the reduced mycobacterial control and survival of coinfected mice was not due to impaired viral clearance and unrestrained IAV infection.

### The Mtb-specific CD4^+^ T cell response is impaired in IAV-coinfected animals.

**Figure 3:**
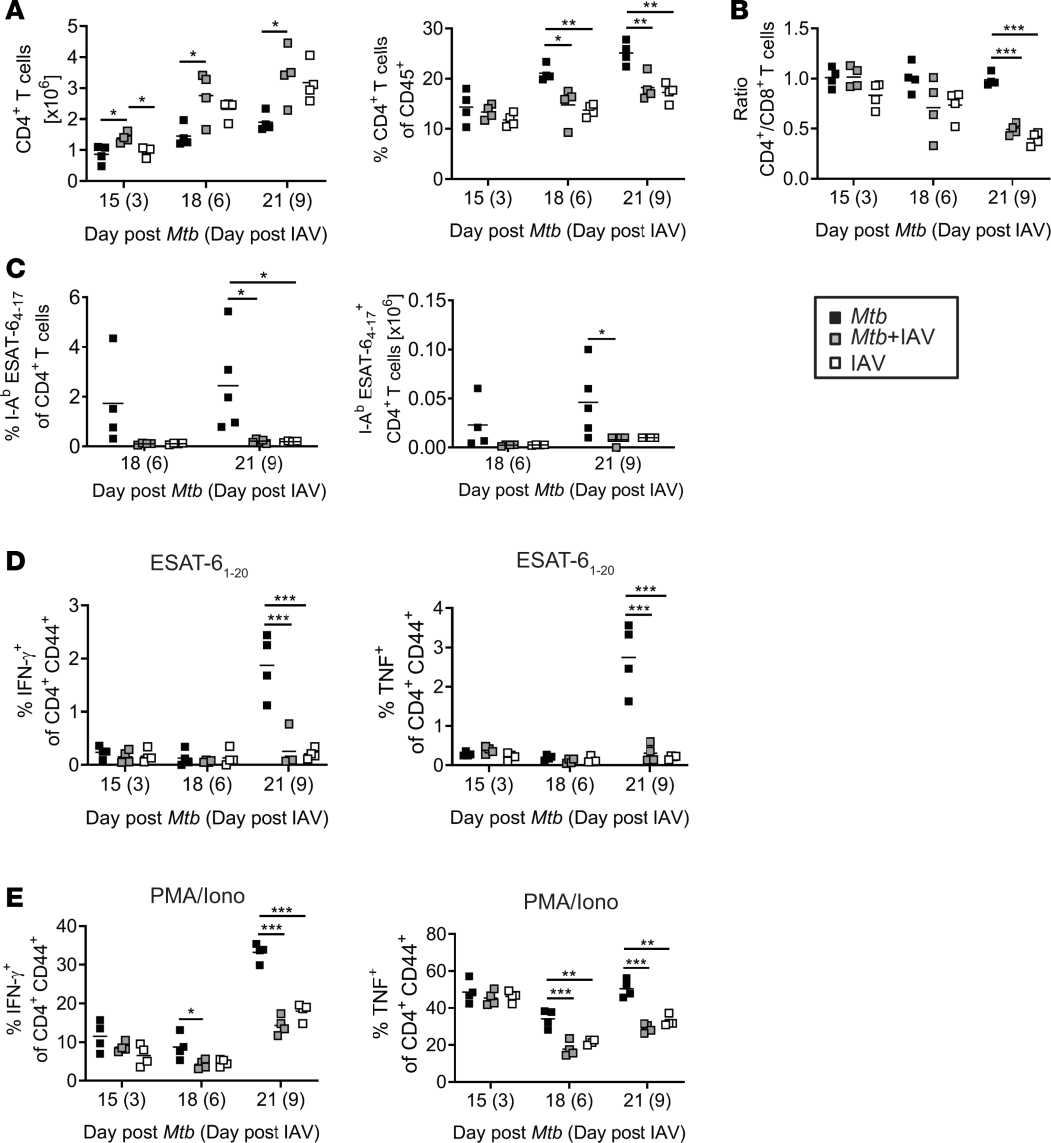
IAV coinfection impairs *Mtb*-specific CD4^+^ T cell response. C57BL/6 mice were infected via aerosol with a low dose of *Mtb* H37Rv and 12 days later coinfected i.n. with 1 × 10^4^ PFU IAV (A/HH/05/09 H1N1). Lungs were analyzed by flow cytometry at indicated time points for (**A**) total CD4^+^ T cell numbers and frequencies among CD45^+^ cells, (**B**) ratio of CD4^+^ to CD8^+^ T cells (**A** and **B**, *n* = 4 mice per group, representative of 2 independent experiments), (**C**) for I-A^b^ ESAT-6_4–17_–specific CD4^+^ T cells (day 18 *n* = 4 per group, 1 experiment; day 21 *n* = 4–5 per group, representative of 2 independent experiments), and (**D** and **E**) IFN-γ– and TNF-producing CD4^+^ T cells upon ex vivo restimulation with (**D**) ESAT-6_1–20_ peptide or (**E**) PMA/Iono (*n* = 4 per group, day 15 and day 18 *Mtb* representative of 2 independent experiments, day 21 *Mtb* representative of 4 independent experiments). Each data point represents 1 mouse. *P ≤ 0.05; **P ≤ 0.01, ***P ≤ 0.001 determined by (**A**–**E**) 1-way ANOVA followed by Tukey’s multiple-comparison test.

Elevated bacterial loads in coinfected lungs indicate that IAV interferes with the *Mtb*-specific T cell response. Although overall numbers of CD4^+^ T cells were significantly higher in coinfected compared with *Mtb-*infected lungs, the proportion of CD4^+^ T cells among CD45^+^ cells was significantly reduced (Figure 3A) because the IAV infection shifted the CD4^+^/CD8^+^ ratio toward CD8^+^ T cells (Figure 3B). To test whether the *Mtb*-specific CD4^+^ T cell compartment was affected by IAV coinfection, we determined the frequency of *Mtb*-specific CD4^+^ T cells in the lungs of single- and coinfected animals using MHC class II tetramers containing the immunodominant *Mtb* epitope ESAT-6_4–17_ recognized by CD4^+^ T cells in C57BL/6 mice. Compared with mice infected with *Mtb* alone, the frequencies and numbers of tetramer-positive CD4^+^ T cells in the lung were reduced 6 and 9 days after IAV coinfection (18 and 21 days after *Mtb* infection; Figure 3C).

IFN-γ and TNF are critically involved in the control of *Mtb* infection (29). Therefore, we next sought to determine the frequency of *Mtb-*specific CD4^+^ T cells producing IFN-γ and TNF. To this end, we restimulated lung single-cell suspensions either polyclonally with PMA/ionomycin (Iono) or specifically with ESAT-6_1–20_ and determined the frequencies of IFN-γ or TNF producers by intracellular cytokine staining (ICS) followed by flow cytometry. Fifteen and 18 days after *Mtb* infection (3 and 6 days after IAV), frequencies of CD4^+^ T cells that produce IFN-γ or TNF in response to ESAT-6_1–20_ were very low and there were no differences between the groups (Figure 3D). After 21 days, we detected increased frequencies of IFN-γ– and TNF-producing CD4^+^ T cells in response to ESAT-6_1–20_ in lungs of *Mtb-*infected mice. However, the ESAT-6_1–20_–specific CD4^+^ T cell responses were significantly decreased in IAV-coinfected mice (Figure 3D). Likewise, polyfunctional restimulation with PMA/Iono revealed a significant reduction in IFN-γ– and TNF-producing CD4^+^ T cells on day 6 after IAV coinfection (Figure 3E), which was even more pronounced on day 9 after coinfection (day 21 *Mtb* infection; Figure 3E).

Together, these data indicate that IAV coinfection impairs *Mtb-*specific CD4^+^ T cell responses and the production of proinflammatory cytokines IFN-γ and TNF both of which are required for the activation of macrophages to control *Mtb* replication (29, 30).

### IAV coinfection interferes with the priming of Mtb-specific CD4^+^ T cells in the lung-draining lymph nodes.

**Figure 4:**
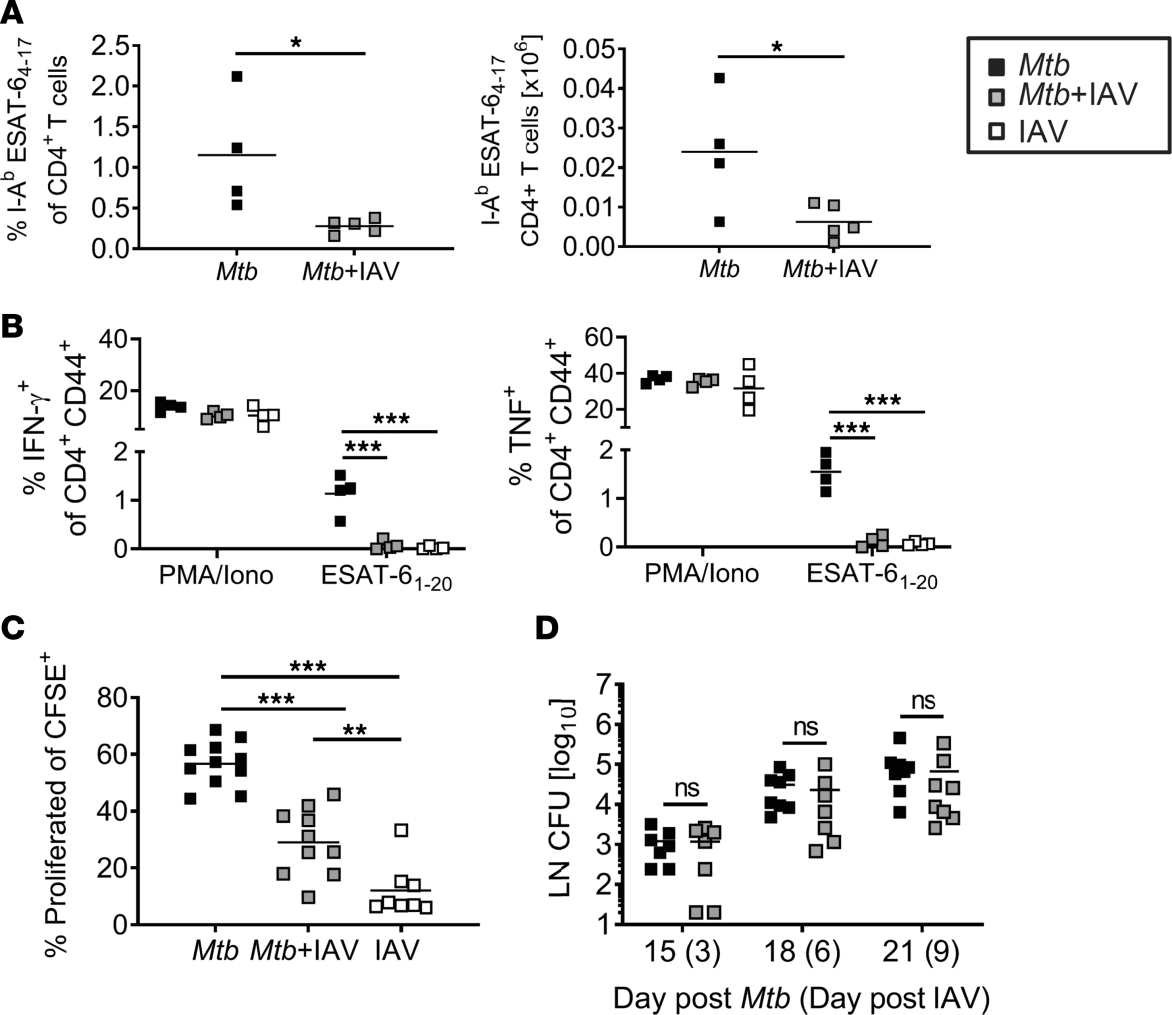
Impaired priming of *Mtb-*specific CD4^+^ T cells in dLNs in coinfected mice. C57BL/6 mice were infected via aerosol with a low dose of *Mtb* H37Rv and 12 days later coinfected i.n. with 1 × 10^4^ PFU IAV (A/HH/05/09 H1N1). dLNs were collected 9 days after IAV (co)infection (day 21 *Mtb*) and analyzed by flow cytometry for the presence of (**A**) I-A^b^ ESAT-6_4–17_–specific CD4^+^ T cells (*n* = 4–5 per group, representative of 2 independent experiments) and (**B**) IFN-γ– and TNF- producing CD4^+^ T cells upon ex vivo restimulation with PMA/Iono or ESAT-6_1–20_ peptide (*n* = 4 per group, representative of 2 independent experiments). (**C**) dLNs from mice that received CFSE-labeled CD4^+^ T cells from P25TCR-tg mice were collected at day 21 *Mtb* (day 9 IAV) and analyzed for the proportion of proliferated CD4^+^ T cells by flow cytometry (*n* = 8–11 per group, representative of 2 independent experiments). (**D**) dLNs were collected at indicated time points and analyzed for bacterial burden (*n* = 7–8 per group, pooled data from 2 independent experiments). Each data point represents 1 mouse. *P ≤ 0.05; **P ≤ 0.01, ***P ≤ 0.001 determined by (**A** and **D**) unpaired *t* test and (**B** and **C**) 1-way ANOVA followed by Tukey’s multiple-comparison test.

Reduced frequencies of ESAT-6–specific CD4^+^ T cells in the lungs during coinfection can be a result of impaired recruitment of *Mtb-*specific T cells from the lung-draining lymph nodes (dLNs) to the lung or of defective T cell priming in the dLNs. We determined the frequency of *Mtb*-specific CD4^+^ T cells in the dLNs of single- and coinfected animals using ESAT-6 tetramer staining as described previously. Like in the lungs, the frequencies and numbers of tetramer-positive CD4^+^ T cells in the dLNs were reduced in *Mtb-*infected mice that were coinfected with IAV for 9 days (Figure 4A). Moreover, the frequencies of CD4^+^ T cells producing IFN-γ or TNF in response to ex vivo restimulation with ESAT-6_1–20_ were significantly reduced when *Mtb-*infected mice were coinfected with IAV (Figure 4B). To further corroborate our findings, we tested whether the activation of CD4^+^ T cells in the dLNs was impaired. To this end, using adoptive cell transfer, we transferred CFSE-labeled P25 T cell receptor-transgenic (P25TCR-tg; *Mtb* antigen 85–specific) CD4^+^ T cells into naive recipients 1 day before *Mtb* infection. On day 9 of coinfection (21 days after initial *Mtb* infection), the frequency of proliferated P25TCR-tg CD4^+^ T cells was significantly lower in the dLNs of coinfected compared with *Mtb*-only-infected mice (Figure 4C), suggesting defective *Mtb*-specific CD4^+^ T cell priming in the presence of IAV.

Initiation of T cell responses to *Mtb* requires the dissemination of bacteria to the dLNs (31, 32). However, we could not detect any significant differences in the numbers of *Mtb* recovered from the dLNs 3, 6, or 9 days after IAV coinfection (Figure 4D).

Taken together, IAV coinfection impaired priming of *Mtb*-specific CD4^+^ T cells in the dLNs, which was not associated with reduced transport of *Mtb* to dLNs but presumably a consequence of the altered immunological environment during coinfection.

### IAV coinfection induces regulatory T cells.

**Figure 5:**
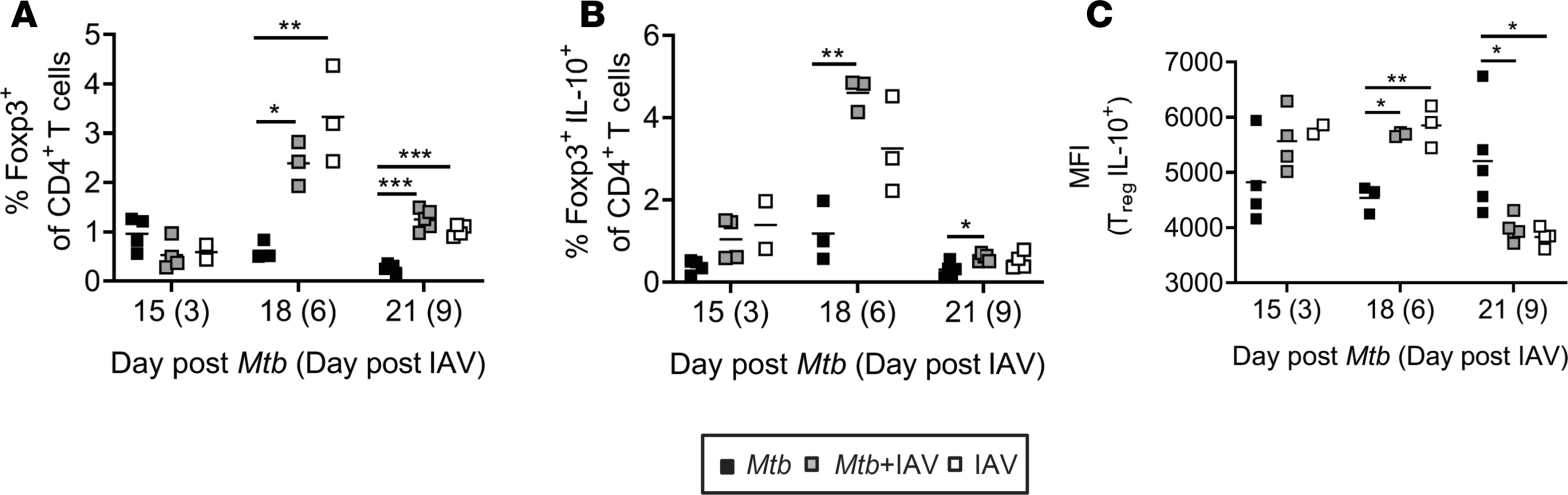
IAV coinfection induces Tregs. C57BL/6 mice were infected via aerosol with a low dose of *Mtb* H37Rv and 12 days later coinfected i.n. with 1 × 10^4^ PFU IAV (A/HH/05/09 H1N1). Lungs were collected at indicated time points and analyzed by flow cytometry for (**A**) presence of Foxp3^+^ CD4^+^ T cells, (**B**) IL-10–producing Foxp3^+^ CD4^+^ T cells and (**C**) the MFI of IL-10 produced by Foxp3^+^ CD4^+^ T cells (*n* = 3–5 per group [day 15 IAV *n* = 2], representative of 2 independent experiments). Each data point represents 1 mouse. *P ≤ 0.05; **P ≤ 0.01, ***P ≤ 0.001 determined by (**A**–**C**) 1-way ANOVA followed by Tukey’s multiple-comparison test.

A subset of CD4^+^ T cells expressing the transcription factor Foxp3, called Tregs, plays a critical role in regulating immune responses. Although Tregs prevent immunopathology, they have been shown to delay the onset of adaptive immunity during the initial T cell response to *Mtb* infection (33). In order to determine whether Tregs are induced during *Mtb-*IAV coinfection, we analyzed their frequencies by flow cytometry. We found that 6 days after IAV coinfection, Tregs in the lungs were significantly elevated compared with *Mtb*-only-infected mice (Figure 5A). Their frequency peaked at day 6 after IAV infection (day 18 *Mtb* infection) but was still significantly increased in coinfected lungs 9 days after IAV infection (day 21 *Mtb* infection) compared with lungs from *Mtb*-alone mice.

Tregs can mediate their suppressor effector function by many different pathways including the release of soluble factors such as IL-10 (34). Polyfunctional ex vivo restimulation followed by ICS revealed that on day 6 and 9 after IAV (co)infection, the frequencies of Foxp3^+^ Tregs producing IL-10 were significantly increased in the lungs of (co)infected compared with *Mtb-*alone-infected mice (Figure 5B). However, Foxp3^+^ Tregs did not produce IL-10 in response to ESAT-6_1–20_ (tested 6 and 9 days after IAV coinfection; data not shown), indicating that polyclonally stimulated IL-10 producers represent IAV-induced Tregs. In addition to a higher percentage of IL-10–expressing Tregs in coinfected lungs, IL-10 producers also showed significantly higher median fluorescence intensity (MFI) for IL-10 on day 6 after IAV (co)infection suggesting higher IL-10 production per cell (Figure 5C).

### IAV coinfection stimulates the production of IL-10 by cells of the innate and adaptive immunity.

**Figure 6:**
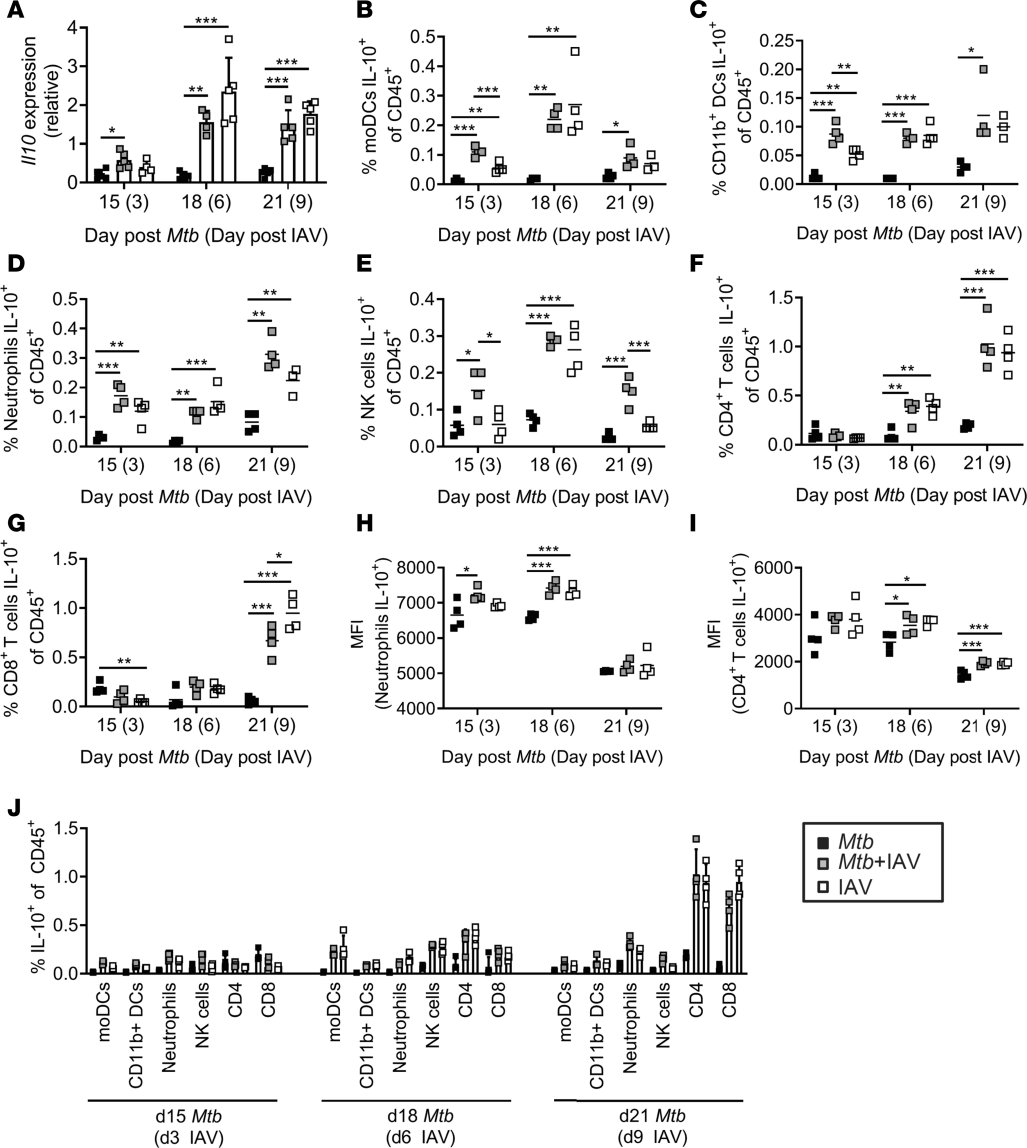
IAV coinfection stimulates the production of IL-10 by innate and adaptive immune cells. C57BL/6 mice were infected via aerosol with a low dose of *Mtb* H37Rv and 12 days later coinfected i.n. with 1 × 10^4^ PFU IAV (A/HH/05/09 H1N1). Lungs were collected at indicated time points and analyzed for (**A**) *Il10* expression by qRT-PCR relative to *Gapdh* expression (*n* = 4–5 per group, representative of 2 independent experiments) and (**B**–**I**) IL-10–producing cells of the innate and adaptive immunity among CD45^+^ cells by flow cytometry with (**B**) moDCs, (**C**) CD11b^+^ DCs, (**D**) neutrophils, (**E**) NK cells, (**F**) CD4^+^ and (**G**) CD8^+^ T cells (*n* = 3–4 per group, data representative of 2 independent experiments). (**H** and **I**) show MFIs of IL-10 producers among (**H**) neutrophils and (**I**) CD4^+^ T cells (*n* = 3–4 per group, data representative of 2 independent experiments). (**J**) Depicts a summary of the frequencies of IL-10^+^ cell types of CD45^+^ per day (*n* = 3–4 per group, data representative of 2 independent experiments). (**A** and **J**) Data are represented as mean + SD or (**B**–**I**) each point representing 1 mouse. *P ≤ 0.05; **P ≤ 0.01, ***P ≤ 0.001 determined by (**A**–**I**) 1-way ANOVA followed by Tukey’s multiple-comparison test.

The observation that IAV coinfection induced Tregs that produce IL-10 prompted us to determine the overall expression of *Il10* in lungs of single- and coinfected mice at different days after IAV coinfection. Quantitative real-time PCR (qRT-PCR) revealed that *Il10* gene expression was low in *Mtb-*infected lungs at all 3 time points analyzed (Figure 6A). By contrast, *Il10* transcripts were significantly upregulated in response to IAV infection in IAV-single and coinfected mice.

*Il10* expression is not restricted to Tregs, but instead it is broadly expressed by many immune cells (35). In order to reveal which cells contributed to its production in coinfected lungs, we performed ICS and flow cytometry upon ex vivo restimulation of lung single-cell suspensions. We detected significantly increased frequencies of IL-10 producers among cells of the innate immune system after polyfunctional ex vivo stimulation. Monocyte-derived dendritic cells (moDCs), CD11b^+^ DCs, neutrophils, and NK cells contributed to IL-10 production 3 days after IAV infection (Figure 6, B–E). In addition to those cells of the innate immunity, CD4^+^ and CD8^+^ T cells contributed with increasing proportions to IL-10 production (Figure 6, F and G) and became the main source of IL-10 by day 9 of IAV coinfection among the cell types analyzed (Figure 6J). For neutrophils and CD4^+^ T cells, significantly higher MFIs for IL-10 in coinfected compared with *Mtb*-alone animals were detected pointing to even higher IL-10 production on a per-cell basis (Figure 6, H and I). None of the cell types responded to ESAT-6_1–20_ restimulation (tested 3, 6, and 9 days after IAV coinfection for moDCs and neutrophils; tested 6 and 9 days after IAV coinfection for CD4^+^, CD8^+^, and NK cells; data not shown) strengthening the assumption that IL-10 production was induced by IAV infection and not in response to *Mtb*.

### IAV coinfection changes macrophage polarization.

**Figure 7:**
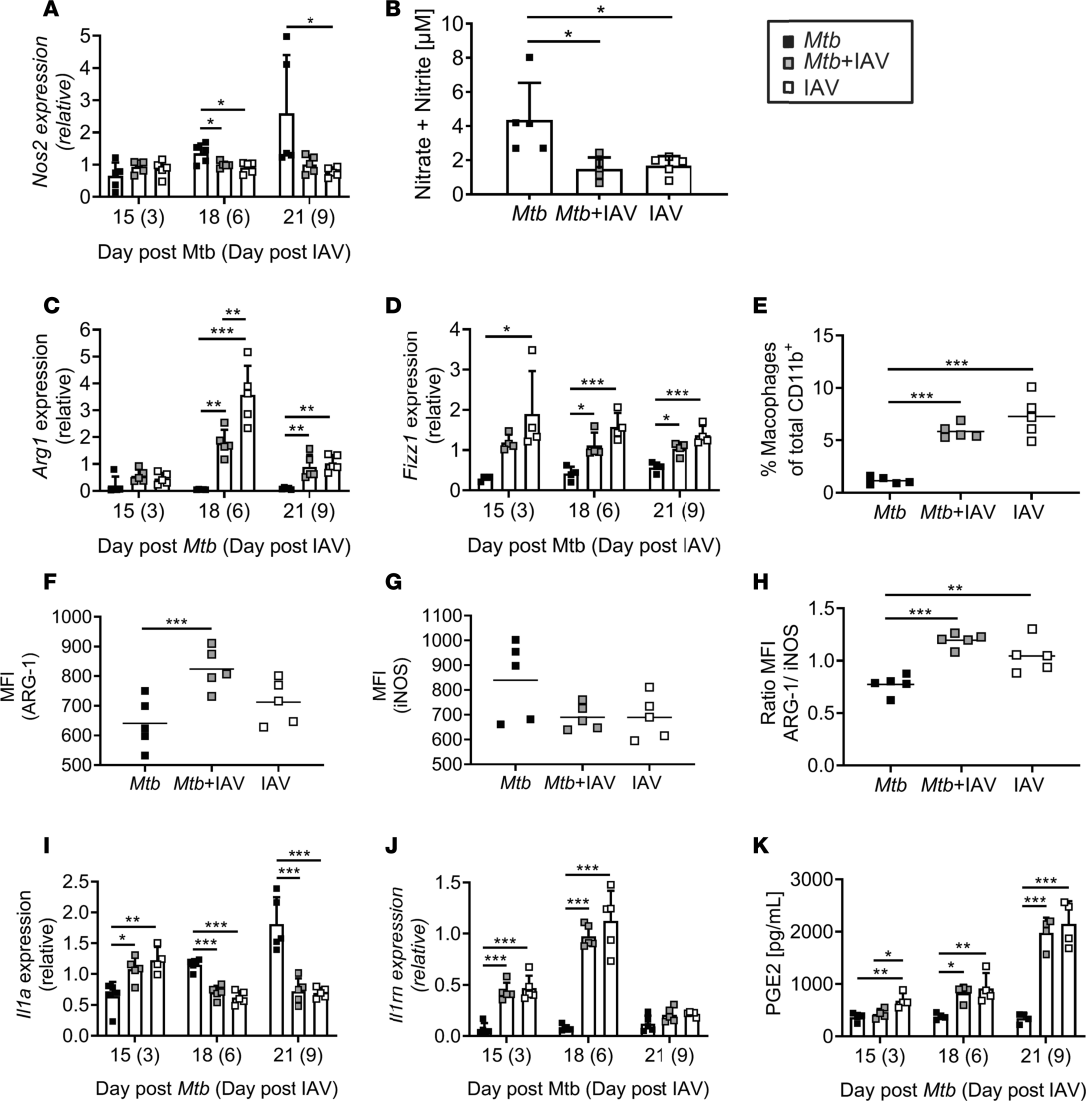
IAV infection changes macrophage polarization. C57BL/6 mice were infected via aerosol with a low dose of *Mtb* H37Rv and 12 days later coinfected i.n. with 1 × 10^4^ PFU IAV (A/HH/05/09 H1N1). Lungs were collected at indicated time points and analyzed for (**A**) *Nos2* expression, (**B**) amount of RNI (*n* = 5 per group, 1 experiment), (**C**) *Arg1* and (**D**) *Fizz1* gene expression. Lungs were collected on day 21 *Mtb* (day 9 IAV) and analyzed by flow cytometry for (**E**) macrophage (CD11b^hi^F4/80^+^Ly6G^–^CD11c^+^Ly6C^+^) frequencies among CD11b^+^ cells (*n* = 5 per group, 1 experiment) and their (**F** and **G**) MFI for (**F**) ARG-1 and (**G**) iNOS (*n* = 5 per group, 1 experiment). (**H**) Shows the ratio of the MFI of ARG-1 to the MFI of iNOS in the defined macrophages. At indicated time points, lung homogenates were analyzed for (**I**) *Il1a* and (**J**) *Il1rn* expression by qRT-PCR or (**K**) the lipid mediator PGE2 by LC-MS^2^ (*n* = 4 per group, representative of 2 independent experiments). Gene expression determined by qRT-PCR relative to *Gapdh* (*n* = 4–5 per group, representative of 2 independent experiments). (**A**–**D** and **I**–**K**) Data are represented as mean + SD or (**E**–**H**) each point representing 1 mouse. *P ≤ 0.05; **P ≤ 0.01, ***P ≤ 0.001 determined by (**A**–**K**) 1-way ANOVA followed by Tukey’s multiple-comparison test.

IL-10 is an antiinflammatory cytokine that can ameliorate immunopathology by limiting innate and adaptive responses involved in tissue damage (36). However, the very same responses are usually required for optimal pathogen clearance. For example, IL-10 inhibits the development of Th1 responses and limits the innate effector functions of macrophages (24). We have shown that *Mtb-*specific CD4^+^ T cell responses are severely impaired in the presence of IAV. Reduced IFN-γ and TNF producers among CD4^+^ T cells together with enhanced expression of IL-10 prompted us to analyze the expression of macrophage activation-associated proteins. As such, nitric oxide synthase 2 (NOS2) is induced by IFN-γ and responsible for the production of reactive nitrogen intermediates (RNIs) required for the killing of *Mtb* (37, 38). qRT-PCR revealed that transcripts for *Nos2* were induced in lungs during *Mtb* infection but induction was significantly reduced when IAV was concurrent (Figure 7A). In line with this finding, we found the production of RNI to be significantly lower in coinfected compared with *Mtb*-only-infected lungs 9 days after IAV coinfection (day 21 *Mtb* infection; Figure 7B). Because IL-10 has been shown to suppress anti-*Mtb* effector mechanisms by the induction of alternatively activated macrophages (AAMs; ref. 39), we next determined the expression of *Arg1* and *Fizz1*, which are signature markers for AAMs (40). Although *Arg1* expression was low in *Mtb-*infected lungs at all time points analyzed, IAV infection induced a significant upregulation in *Arg1* transcripts 6 days after coinfection (Figure 7C). Expression levels dropped by day 9 of IAV coinfection but remained significantly elevated compared with *Mtb*-only-infected lungs. Likewise, we found *Fizz1* expression to be rapidly increased reaching significantly elevated levels in IAV-(co)infected compared with *Mtb*-infected mice 6 and 9 days after IAV infection (Figure 7D).

Increased expression of AAM signature markers prompted us to more specifically analyze macrophages by flow cytometry. Strikingly, we found significantly increased frequencies of macrophages (CD11b^hi^F4/80^+^Ly6G^–^CD11c^+^Ly6C^+^) (41–43) in IAV-(co)infected lungs compared with *Mtb*-infected lungs on day 21 after *Mtb* infection (Figure 7E). In accordance with our qRT-PCR data, macrophages from coinfected mice showed a significantly higher MFI for arginase 1 (ARG-1) indicating an increased production on a single-cell basis (Figure 7F), while inducible nitric oxide synthase (iNOS/NOS2) was decreased (Figure 7G) resulting in an inversed ARG-1/iNOS ratio in coinfected compared with *Mtb*-only-infected mice (Figure 7H). Together, these data strongly suggest that IAV coinfection is accompanied by macrophage polarization toward an AAM phenotype, which is associated with reduced antimycobacterial effector mechanisms (39, 44, 45).

In addition to RNI, IL-1 has also been shown to play a major role in myeloid-mediated protection against *Mtb* (18). Both type I IFNs and IL-10 can directly inhibit IL-1 expression and induce the IL-1R antagonist (IL-1RA) (23). The induction of type I/III IFNs (Figure 2A) and IL-10 (Figure 6) in IAV-coinfected mice therefore prompted us to analyze the expression of *Il1a* and *Il1rn*. Twenty-one days after *Mtb* infection, *Il1a* transcripts were significantly reduced in the presence of IAV (Figure 7I). In contrast, *Il1rn* was rapidly induced in IAV-(co)infected lungs and significantly elevated compared with *Mtb*-only-infected lungs (Figure 7J). In conclusion, our data implicate impaired IL-1 signaling when mice are coinfected with IAV.

IL-1–mediated protection in *Mtb* infection largely depends on the production of the lipid mediator prostaglandin E2 (PGE2), which has been shown to limit excessive type I IFN production (22). While virulent *Mtb* can inhibit PGE2 production, it is induced during IAV infection where it might interfere with viral clearance (46). We therefore sought to determine PGE2 production in the context of *Mtb*-IAV coinfection and found that IAV coinfection markedly increased PGE2 in *Mtb-*infected lungs (Figure 7K), suggesting an IL-1–independent production in response to IAV infection.

These data collectively indicate that concurrent IAV infection induces an environment associated with impaired innate antimycobacterial effector functions.

### Anti–IL-10 receptor treatment reduces Mtb load in coinfected mice.

**Figure 8:**
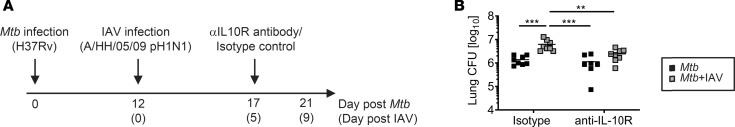
Anti-IL10R treatment reduces *Mtb* load in coinfected mice. C57BL/6 mice were infected via aerosol with a low dose of *Mtb* H37Rv and 12 days later coinfected with 1 × 10^4^ PFU IAV (A/HH/05/09 H1N1). (**A**) Timeline of experimental setup. Two hundred micrograms of anti-IL10R antibody (1B1.3) or isotype control (polyclonal rat serum IgG) was administered i.p. on day 5 after IAV infection (day 17 *Mtb*). (**B**) Lungs were collected at day 21 *Mtb* (day 9 IAV) and analyzed for bacterial burden (*n* = 7–8 per group, pooled data from 2 independent experiments). Each data point represents 1 mouse. **P ≤ 0.01, ***P ≤ 0.001 determined by (**B**) 2-way ANOVA followed by Tukey’s multiple-comparison test.

The fact that we found impaired *Mtb*-specific CD4^+^ T cell responses and evidence for AAMs concomitant with increased *Il10* expression in coinfected mice prompted us to determine the role of IL-10 receptor (IL-10R) signaling in influenza-induced exacerbation of *Mtb* infection. To this end, we treated mice once with an anti–IL-10R monoclonal antibody on day 5 after IAV coinfection (day 17 *Mtb* infection; Figure 8A) and determined the lung CFU another 4 days later (day 21 *Mtb* infection). Although treatment with an isotype control had no effect on the increased mycobacterial load, blocking IL-10R signaling significantly reduced the bacterial load in coinfected lungs to a level comparable to *Mtb-*single-infected animals (Figure 8B). Surprisingly, anti–IL-10R treatment of coinfected mice did not restore *Mtb*-specific CD4^+^ T cell responses in the lung (Supplemental Figure 2, A–C), indicating that impaired control of *Mtb* during IAV coinfection was T cell independent.

Together, our data suggest that IL-10/IL-10R signaling constitutes a major pathway that enhances susceptibility to *Mtb* during concurrent IAV infection.

## Discussion

Recent epidemiological and experimental findings point to an association between Tb and influenza virus infection and increased morbidity and mortality (4, 6, 7, 11). Whereas an influenza virus infection could render individuals more susceptible to *Mtb*, as is the case for other secondary bacterial superinfections, exposure of individuals with an already established *Mtb* infection to influenza viruses could result in reactivation of latent or exacerbation of active Tb. In fact, recent studies found influenza virus infection to be associated with an increased risk of death among patients with pulmonary Tb (5, 6), and the WHO raised concerns about the possible impact of influenza on patients with active Tb during the 2009 H1N1 influenza pandemic (4). A recent small-scale study from The Gambia revealed that around 4% of patients were coinfected with influenza viruses at the time of Tb diagnosis. Strikingly, coinfected patients had a significantly higher bacterial load in sputum compared with those with Tb infection alone, indicating that influenza infection interferes with the host immune response to control *Mtb* infection (47). In the present study, we experimentally addressed the question how subsequent IAV infection challenges the control of a pre-existing *Mtb* infection in C57BL/6 mice. IAV coinfection was associated with significantly increased bacterial loads, reduced survival, and a substantial modulation of innate and adaptive immune defenses. Our findings strongly indicate that IAV coinfection compromises the host’s ability to control *Mtb* infection via the production of IL-10, which was rapidly induced upon viral infection. Accordingly, blockade of IL-10R signaling was able to reduce the increased bacterial load in coinfected mice to a level comparable to *Mtb-*single-infected animals. While our study yet again supports a detrimental role of IL-10 during *Mtb* infection, we show for the first time that it is a major driver of IAV-mediated disease exacerbation.

In our coinfection model, IL-10 was not markedly expressed in response to *Mtb* infection alone but rather rapidly induced following IAV coinfection. This is in line with other studies showing resistant C57BL/6 mice to produce lower amounts of IL-10 in response to *Mtb* infection and only at later time points than susceptible strains such as CBA/J (48, 49). In contrast, IL-10 is strongly induced during IAV infection where it counterbalances exuberant inflammation and prevents lethal injury (50, 51). IL-10 can be produced by multiple cells of the innate and the adaptive immune system (35, 52). Our data suggest that innate and adaptive immune cells contribute to IAV-induced IL-10 production, with CD4^+^ T cells being the main source of IL-10. Importantly, IL-10 production by T cells was stimulated polyclonally but not by ESAT-6_1–20_, further suggesting that in coinfected mice, IL-10 is produced in response to IAV and not to *Mtb*.

Overproduction of IL-10 is known to have detrimental effects on *Mtb* infection. IL-10 has previously been shown to interfere with Th1 cell and macrophage function (39, 48, 49, 53–56), and IL-10 deficiency improves the outcome of *Mtb* infection mainly due to enhanced macrophage and Th1 responses (49, 55, 57). In mice coinfected with IAV, increased IL-10 production was concomitant with impaired *Mtb*-specific CD4^+^ T cell responses. Our data are in line with another experimental study by Flórido et al. who reported that simultaneous IAV infection impaired mycobacteria-specific T cell responses and mycobacterial clearance from the lungs using the attenuated Tb vaccine strain *M*. *bovis* Bacillus Calmette-Guérin (58). However, although we found increased IL-10 production in IAV coinfected animals concurrent with impaired *Mtb*-specific T cell responses, anti–IL-10R treatment did not restore *Mtb*-specific T cell responses in the lungs of coinfected mice. Nevertheless, a single injection of anti–IL-10R antibody 4 days before CFU analysis sufficed to reduce pulmonary bacterial loads to levels comparable to those in *Mtb*-single infected mice. These findings indicate that IL-10 contributed to impaired control of *Mtb* during IAV coinfection but in a T cell independent manner.

It was shown before that mice with T cell or macrophage-specific overexpression of IL-10 were highly susceptible to mycobacterial infections without compromised T cell immunity (39, 54, 56). Instead, IL-10 compromised the antimycobacterial effector functions of macrophages. Schreiber et al. found that animals with macrophage-specific overexpression of IL-10 showed signs of alternative macrophage activation and IL-10–dependent ARG-1 induction diminished antimycobacterial effector mechanisms in macrophages (39). Likewise, mice coinfected with IAV showed an increased frequency of macrophages, which showed higher ARG-1 production, and increased expression of *Arg1* together with decreased expression of *Nos2* were accompanied by reduced RNI production. In a different coinfection model, helminth infection resulted in a transitory impairment of resistance to airborne *Mtb* infection (44). Interestingly, helminth infection did not impair protective *Mtb*-specific T cell responses, but the helminth-induced Th2 response caused the accumulation of AAMs in the lung. Because AAMs support the intracellular persistence of *Mtb* (45, 59), they may provide a niche for *Mtb* survival and replication. Therefore, our findings suggest that IAV coinfection detrimentally affects the outcome of *Mtb* infection by promoting the accumulation of a permissive macrophage population in the lungs. The fact that we found decreased expression of *Il1a* also indicates impaired macrophage activation. Moreover, because IL-1α is required for host resistance to *Mtb* (18), its reduced expression may contribute to impaired control of *Mtb* in coinfected mice.

The induction of IL-10 during IAV infection can be promoted by type I IFNs or PGE2 (52). Considering the kinetics of the induction of IL-10, type I IFNs, and PGE2, we found IFN induction but not PGE2 to precede the peak in IL-10 production, indicating that IL-10 expression could at least in part be mediated downstream of type I IFNs. With decreasing expression of IFNs, the production of PGE2 steadily increased in IAV-coinfected mice while there was no overt production in *Mtb-*alone mice. PGE2 has been shown to inhibit type I IFN expression during IAV infection (46) thereby impairing effective antiviral immunity. In our study, however, PGE2 gradually increased over time with peak production on day 9 after IAV infection, when viral infection was already under control and mostly cleared. This might explain why we did not observe impaired viral clearance in our coinfection model and suggests a role for PGE2 in resolution of IAV infection (60). On the other hand, PGE2 is considered beneficial during *Mtb* infection because it protects against *Mtb* infection-induced necrosis (61, 62). The positive role of PGE2 for *Mtb* control has been shown in *Ptges*-deficient macrophages and mice that harbor increased bacterial burden (61). Despite greatly elevated levels of PGE2 in coinfected lungs 9 days after IAV infection (day 21 post *Mtb* infection), mycobacterial loads were significantly increased compared with *Mtb*-only-infected mice. Importantly, PGE2 has significant immunosuppressive effects (63), and high levels of PGE2 during late-stage *Mtb* infection were shown to inhibit bacterial killing, nitrate production, and Th1 cytokines (64). Thus, downstream effects elicited by PGE2 depend on its concentration, and the substantial increase in PGE2 levels during IAV coinfection might counteract antimycobacterial effector functions. In good agreement are observations from a peritonitis model of acute inflammation, where resolution triggered a prolonged phase of PGE2-mediated immune suppression that reduced local innate immunity to bacterial infection and inhibited lymphocyte function (65). Downmodulation of proinflammatory immune responses by PGE2 can be due to the upregulation of *Il10* expression (66–69). As discussed previously, however, the kinetics of the induction of IL-10 and PGE2 suggest that IL-10 production was induced independently of PGE2.

In order to confirm the role of IL-10 in IAV-induced exacerbation of *Mtb* infection, we blocked IL-10R signaling during coinfection by antibody treatment. A single application was sufficient to reduce mycobacterial loads in coinfected animals to a level comparable to those in *Mtb*-only-infected mice. Thus, the impaired control of *Mtb* during IAV coinfection was most likely a consequence of increased IAV-induced IL-10 production. The importance of controlling IL-10 production during *Mtb* infection has been demonstrated very recently by Huynh et al. who found the transcription factor basic helix-loop-helix family member e40 to repress *Il10* expression during *Mtb* infection in C57BL/6 mice (70). Thus, while the production of IL-10 in response to *Mtb* is controlled on a transcriptional level, exogenous factors that lead to increased *Il10* expression can render normally resistant animals susceptible to *Mtb*.

IL-10 can have different roles during IAV infection. On the one hand, IL-10 controls lung inflammation and prevents lethal injury (51). On the other hand, it can also play a detrimental role, as in the absence of IL-10, viral clearance, and survival in mice was improved (71, 72). Differences in the outcome of IL-10 deficiency could, in part, be explained by different virus strains or doses of infection and the time of IL-10 blockade. The viral strain used in our study is only low pathogenic in C57BL/6 mice and does not cause severe lung damage (25). This might explain why blocking IL-10R signaling did not aggravate IAV infection in our model.

Current research strategies targeting host factors, rather than pathogen components directly, aim at the development of novel treatment approaches termed host-directed therapies (HDTs). Such approaches could augment protective immunity and potentiate antimycobacterial effector mechanisms to reduce the duration of Tb antimicrobial therapy. Given its detrimental role during Tb, IL-10 is an attractive target for HDT. An HDT approach applying aerosolized peptide inhibitors targeting the IL-10R alpha chain had major beneficial effects on *Mtb* control in C57BL/6 mice (73). Future studies need to elucidate whether such treatment could also be applicable in situations of coinfection-induced disease exacerbation.

Our findings may have implications for the management of human *Mtb* infection and emphasize the need to tackle flu infections in areas with high Tb prevalence either by vaccination or prophylactic flu treatment. In light of a recent study that reported influenza vaccination can reduce the incidence of Tb in the elderly (74), our data strongly suggest that influenza vaccination should be considered for risk groups in areas with high Tb prevalence. Reducing the incidence or severity of Tb by targeting a potential risk factor such as IAV infection would contribute to reduced antibiotic use and may slow down the development and spread of resistance (1).

Our study confirms the negative impact of concurrent IAV on *Mtb* infection described previously (11) and identifies IL-10 as major player in Tb disease exacerbation during IAV coinfection. Our findings underline the importance to not only understand immune responses and disease pathogenesis for pathogens individually but to further examine coinfections as risk factors for Tb exacerbation.

## Methods

### Mice.

All mice used were bred under specific pathogen–free conditions at the Research Center Borstel and maintained under specific barrier conditions either in the BSL-3 or BSL-2 facility at the Research Center Borstel. Male C57BL/6J mice aged between 8 to 12 weeks and female and male P25TCR-tg mice on a C57BL/6 background (75) aged between 12 to 16 weeks were used.

### Mtb infection and determination of bacterial load.

*Mtb* H37Rv was grown in Middlebrook 7H9 broth supplemented with 10% v/v OADC (oleic acid, albumin, dextrose, catalase) to logarithmic growth phase (OD600 0.2–0.4), and aliquots were frozen at –80°C.

For infection of mice, *Mtb* stocks were diluted in sterile distilled water at a concentration providing an uptake of 100 to 200 viable bacilli per lung or approximately 2000 for high-dose infection, respectively. Infection was performed via the respiratory route by using an aerosol chamber (Glas-Col). The inoculum size was quantified 24 hours after infection, and bacterial loads in lungs, dLNs, and spleens at indicated time points by determining CFUs as described earlier (76). A clinical score was used to indicate severity of disease progression as described previously (77).

### IAV infection and determination of viral load.

The 2009 pH1N1 WT virus A/Hamburg/05/09 isolated on April 28th from pharyngeal swabs of a female patient prior to oseltamivir treatment as described previously (78) was propagated on Madin-Darby canine kidney cells (MDCK) in the presence of tolylsulfonyl phenylalanyl chloromethyl ketone trypsin (TPCK) (Sigma-Aldrich). Aliquots of virus-containing supernatants were frozen at –80°C. For infection of experimental animals, mice were anesthetized and inoculated i.n. with 50 μl of 1 × 10^4^ plaque-forming units (PFUs) of infectious virus diluted in PBS. Virus titers in lung homogenates were determined by MDCK plaque assay (overlay: 1.25% Avicel RC-581 [IMCD GmbH & Co KG], containing 1 μg/ml TPCK). After 72 hours at 37°C, 5% CO_2_ overlay medium was removed, cells were fixed, permeabilized, and immunostained for IAV nucleoprotein (1° Ab: 9G8, abcam; 2° Ab: HRP-conjugated rabbit anti-mouse IgG [Southern Biotech]; TrueBlue Peroxidase Substrate, KPL Immunoassay Reagents & Kits).

### Measurement of type I and III IFN activity.

To determine the amount of IFN I/III, IFN-sensitive epithelial cells from Mx2-Luc reporter mice were treated with the supernatant as described previously (79). A standard curve for the calculation of IFN activity was obtained by treating cells with serial dilutions of IFN-β.

### Adoptive T cell transfer.

For adoptive transfer experiments, CD4^+^ T cells from spleens of P25TCR-tg mice were magnetically isolated using the CD4^+^ T cell isolation kit (Miltenyi Biotec) according to the manufacturer’s instructions. Isolated CD4^+^ T cells were labeled with CFSE (CFDA-SE, Invitrogen) and 3–5 × 10^6^ cells were adoptively transferred i.v. 24 hours before *Mtb* infection.

### Flow cytometry.

Mice were sacrificed and perfused intracardially with sterile PBS before organ harvest. Single-cell suspensions of lungs were prepared as described previously (80). Lymph nodes were passed through a 100-μm pore size cell strainer. For flow cytometric analysis of surface antigens, intracellular cytokines, and transcription factor, single-cell suspensions were stained with the following specific antibodies (BioLegend unless otherwise stated): APC anti-CD3 (17A2), APC-Cy7 anti-CD3 (17A2), PerCP-Cy5.5 anti-CD4 (RM4-5), BV510 anti-CD4 (RM4-5), APC-Cy7 anti-CD8 (53-6.7), PerCP-Cy5.5 anti-CD44 (IM7), BV510 anti-CD45 (30-F11), BV421 anti-CD45 (30-F11), APC e780 anti-CD90.2 (53-3.1, eBioscience), BV510 anti-CD11b (M1/70), PerCP-Cy5.5 anti-CD11b (M1-70), BV421 anti-CD11c (N418), PE anti-CD11c (N418), BV421 anti-F4/80 (BM8), APC anti-Ly6C (HK1.4), PE-Cy7 anti-Ly6C (HK1.4), PerCP-Cy5.5 anti-Ly6G (1A8), APC-Cy7 anti-Ly6G (1A8), BV421 anti-NK1.1 (PK136), AF647 anti-Fox3 (MF-14), APC anti–IFN-γ (XMG1.2), PE anti-IL10 (JES5-16E3), FITC anti-iNOS/NOS type II (6; no. 610330 BD Bioscience), BV421 anti-TNF (MP6-XT22), anti-arginase I (V-20, Santa Cruz Biotechnology), and DyLight650 donkey F(ab′)2 anti-goat IgG (ab98517, abcam).

For intracellular staining, cells were stimulated 5 hours with PMA/Iono (Cell Activation Cocktail w/o Brefeldin, BioLegend) or 30 μg/ml of C-terminal–modified ESAT-6_1–20_ peptide (MTEQQWNFAGIEAAASAIQG*GXKXK*; Structural Biochemistry, Research Center Borstel) where indicated in the presence of Monensin and Brefeldin A (BioLegend) at 37°C, 5% CO_2_. Nonspecific antibody binding was blocked by incubation with a cocktail containing anti-CD16/32 mAb (BioLegend), mouse, hamster, and rat serum. Cells were surface stained, fixed, and permeabilized (Fixation buffer, Intracellular Staining Permeablization buffer, BioLegend) and stained with indicated antibodies directed against intracellular antigens. For detection of the Foxp3 transcription factor, the True-Nuclear Transcription Factor buffer set (BioLegend) was used according to the manufacturer’s recommendations. Staining of arginase followed a 2-step protocol applying unlabeled anti-arginase 1 prior to incubation with DyLight650 donkey F(ab′)2 anti-goat IgG.

For detection of *Mtb* antigen-specific T cells, single-cell suspensions were incubated with BV421 conjugated tetramer of I-A(b) *Mtb* ESAT6_4–17_ (QQWNFAGIEAAASA; National Institute of Health Tetramer Core) at a final dilution of 1:200 for 1 hour and15 minutes at 37°C, 5% CO_2_ before surface staining. IAV-specific CD8^+^ T cells were detected by staining with H1N1 NP366 Dextramer (ASNENVETM, Immudex) as described earlier (81). Cells were fixed with 2% paraformaldehyde for 20 minutes at 4°C before acquisition. Data were acquired on a FACSCantoII flow cytometer (BD Biosciences) and analyzed with the FCS Express software (DeNovo Software). Gating strategies are depicted in Supplemental Figures 3–10.

### Lipid extraction and liquid chromatography–tandem mass spectrometry analysis.

Lipid extraction was performed according to a modified acidified Bligh and Dyer protocol as described earlier (82). Detailed information can be found in Supplemental Table 1. Organic phases were combined and dried under a slight stream of nitrogen. Lipid extracts were dissolved in 40 μl of solvent A and 2 μl was injected for the chromatography–tandem mass spectrometry (LC-MS^2^) analysis. Eicosanoids analysis was performed with a micro-LC 1100 system (Agilent Technologies) coupled to a Q Exactive Plus mass spectrometer (Thermo Fisher Scientific) as described earlier (83). Details of the chromatographic method and mass analysis are provided in Supplemental Table 1.

Mass spectrometry data interpretation (peak detection and integration) was performed with Xcalibur software (Thermo Fisher Scientific). Transitions for each lipid mediator (LM) for the parallel reaction monitoring method are summarized in Supplemental Table 1. LM identifications were verified with the earlier-reported scoring algorithm (82).

### qRT-PCR.

Total RNA from lung tissue was extracted from peqGOLD Trifast using the Direct-zol RNA MiniPrep Kit (Zymo Research) according to the manufacturer’s protocol. Isolated RNA was reverse transcribed according to the manufacturer’s instructions (Maxima First Strand cDNA Synthesis Kit for RT-qPCR, Life Technologies). qRT-PCR was performed using LightCycler 480 SYBR Green I Master (Roche) on the LightCycler 480 instrument. Analysis of the relative changes was performed using LightCycler480 Software 1.5.0 SP4 (version 1.5.0.39, Roche). All quantifications were normalized to the level of Gapdh gene expression. The following primers were used: *Gapdh* forward ATTGTCAGCAATGCATCCTG, reverse ATGGACTGTGGTCATGAGCC; *Arg1* forward ACAAGACAGGGCTCCTTTCAG, reverse CTGTGATGCCCCAGATGGTT; *Fizz1* forward ATGAACAGATGGGCCTCCTG, reverse TCTTAGGACAGTTGGCAGCA; *Il1a* forward CGCTTGAGTCGGCAAAGAAATC, reverse GTGCAAGTCTCATGAAGTGAGC; *Il1rn* forward TGTGCCAAGTCTGGAGATGA, reverse TTCTTTGTTCTTGCTCAGATCAGT (22); *Il10* forward GGTTGCCAAGCCTTATCGGA, reverse ACCTGCTCCACTGCCTTGCT; *Mx1* forward ATGGGTGAACTCAGGCAATCTC, reverse TTGACAGTCTCCTGCTTAGTGAC; *Nos2* forward CTGCAGCACTTGGATCAGGA, reverse TCCTTTGAGCCCTTTGTGCT.

### NO assay.

To detect RNI, lung homogenates were deproteinated using Micron YM-30 centrifugal filters (Millipore), and the flow-through was analyzed after reductase treatment according to the manufacturer’s protocol (Nitrate/Nitrite Colorimetric Assay Kit, Cayman Chemical). Photometric measurement followed reading the absorbance at 540 nm on a BioTek Synergy 2 microplate reader.

### Blockade of anti-IL10 receptor.

For antibody treatment, mice were injected i.p. once with 200 μg anti–IL-10 receptor-antibody (clone 1B1.3; provided by Rudolf Manz, University of Lübeck, Lübeck, Germany) or 200 μg polyclonal rat serum IgG (Sigma-Aldrich) on day 5 after influenza virus infection.

### Statistics.

First, data were tested for normal distribution using the Shapiro-Wilk test. If data were normally distributed, unpaired Student’s *t* test (for comparison of 2 groups) or 1-way ANOVA (comparison of 3 or more groups) followed by Tukey’s multiple-comparison test was performed. All tests were 2-tailed at the significance level *P* < 0.05. To examine the influence of 2 different independent variables, a 2-way ANOVA followed by Tukey’s multiple-comparison test was performed. A log-rank test (Mantel-Cox) was used for survival analysis. All data were analyzed using GraphPad Prism 8 (GraphPad Software, Inc.).

### Study approval.

All animal experiments were conducted according to German animal protection law. Animal experiments were approved by the Ethics Committee for Animal Experiments of the Ministry of Energy, Agriculture, Environment, and Rural Areas of the State of Schleswig-Holstein.

## Author contributions

BES, SR, and GG conceived and designed the experiments. SR, LE, and AW performed the experiments. SR, JB, AW, DS, AMH, and AK analyzed the data. AMH and CH provided material. BES and SR wrote and edited the manuscript. All coauthors edited and commented on the final manuscript.

## Supplementary Material

Supplemental data

## Version 1 04/18/2019

In-Press Preview

## Version 2 05/16/2019

Electronic publication
