# Supplementary material for: Blocking IL-10 receptor signaling ameliorates Mycobacterium tuberculosis infection during influenza-induced exacerbation
Source: JCI Insight. 2019 May 16;4(10):e126533. doi: 10.1172/jci.insight.126533 (PMC6542649; doi:10.1172/jci.insight.126533)
Supplement: Supplemental data [file jciinsight-4-126533-s232.pdf]

Table S1: Selected parameters for the characterization of LM during an LC-MS^2 run.

Eicosanoids/ Docosanoids	IS	Lipid species	RT (min)	Precursor ion (m/z)	Product ion (m/z)	(N) CE
PUFA-O3	RvD2-d5		2.73	380.2480	175.0762	23
PG	PGE2-d9		2.43	360.2731	189.1274	33
		6-keto-PGE1α	1.56	367.2126	143.0704	23
		RvE1	1.83	349.2020	195.1027	22
		TBX2	1.88	369.2283	195.1028	22
		20-OH-LTB4	1.89	351.2177	195.1016	22
		LxA5	2.03	349.2020	115.0401	22
		PGE3	2.25	349.2020	269.1909	22
		PGF2alpha	2.30	353.2333	247.2062	35
		PGE2	2.47	351.2177	175.1121	22
		LxB4	2.72	351.2177	221.1183	22
		PGI2	2.90	351.2177	215.1070	22
		PGD2	2.81	351.2177	233.1177	22
		RvD2	3.03	375.2177	175.0761	22
		LxA4	3.14	351.2177	115.0401	22
		15-keto-PGE2	3.15	349.2020	113.0957	22
		RvD1	3.32	375.2177	233.1548	22
		PGB2	3.38	333.2071	175.1028	22
		13,14-DiH-15k-PGE2	3.77	351.2177	209.1175	22
		13,14-DiH-15k-PGD2	4.55	351.2177	207.1017	22
PUFA-O2	LTB4-d4		7.39	339.2468	197.1142	25
		5,15-DiHETE	7.01	335.2228	115.0385	25
		17,18-DiHETE	7.10	335.2228	247.1698	25
		trans-LTB4	7.20	335.2228	195.1016	25
		7-Mar1	7.35	359.2228	250.1387	22
		12-epi-LTB4	7.50	335.2228	195.1016	25
		LTB4	7.53	335.2218	195.1016	25
		epi-LTB4	7.75	335.2228	195.1016	25
		PD1	7.82	359.2228	153.0908	22
		14,15-DiHETE	8.5	335.2228	207.1387	25
		5,6-DIHETE	10.48	335.2228	145.0501	25

		12-oxo-LTB4	10.90	333.2071	179.0702	22
PUFA-O1	5-HETE-d8		33.40	327.2770	116.0448	21
		18-HEPE	22.30	317.2122	259.1700	22
		15-HEPE	27.94	317.2122	219.1383	22
		12-HEPE	31.18	317.2122	179.1065	22
		13-HODE	32.26	295.2279	195.1401	30
		9-HODE	32.45	295.2279	171.1015	30
		15-HETE	32.90	319.2279	219.1394	22
		17,18-EpETE	32.97	317.2122	215.1706	22
		17-HDoHE	33.10	343.2279	245.1542	22
		13-HDoHE	33.20	343.2279	193.1233	22
		10-HDoHE	33.29	343.2279	153.0908	22
		14-HDoHE	33.32	343.2279	205.1226	22
		8-HETE	33.33	319.2279	155.0715	22
		12-HETE	33.37	319.2279	179.1067	22
		7-HDoHE	33.41	343.2279	141.0544	22
		5-HETE	33.49	319.2279	115.0401	22
		4-HDoHE	33.67	343.2279	101.0228	22
		12-oxo-ETE	33.96	317.2122	237.1318	22
		5-oxo-ETE	33.96	317.2122	203.1806	22
		14,15-EET	33.97	319.2279	175.1480	22
		11,12-EET	34.20	319.2279	167.1078	22
		5,6-EET	34.25	319.2279	191.1803	22
		8,9-EET	34.29	319.2279	123.0801	22
PUFA	AA-d11		35.65	314.3009	270.3120	21
		EPA	35.10	301.2173	257.2273	22
		DHA	35.45	327.2329	283.2430	21
		AA	35.70	303.2329	259.2430	22
		LA	36.08	279.2326	127.0750	40

Lipid Extraktion

500µl of lung homogenates were transferred into an ice-cooled 2.0 ml tube covered with aluminum foil. Afterwards, 0.5 µl of butyl hydroxyl-toluene in methanol (BHT; 1.0 mg/ml) followed by 25µl of internal standard mix containing PGE2-d9, RvD2-d5, LTB4-d4, 5-HETE-d8, AA-d11 at a concentration of 500 fmol/µL was added. Subsequently, 320 µL of ice-cold chloroform was added together with 640 µL of a freshly prepared ice-cold MeOH/HAc (97+3; v/v) solution to the mixture. The mixture was incubated for 30 min at room temperature under slight stirring. For phase separation 320 µL of ice-cold water was added and the mixture was incubated for another 30 min under constant shaking. At last, the samples were centrifuged at 15,000 x g for 15 min at 4 °C to improve phase separation. Afterwards the organic phase was collected and transferred to an ice-cooled sample tube. The aqueous was three more times extracted with 320 µL of ice-cold $CHCl_3$. Organic phases were combined and dried under a slight stream of nitrogen. Lipid extracts were dissolved in 40 µL of solvent A and 2 µL were injected for the LC-MS^2 analysis.

Liquid chromatography – tandem mass spectrometry (LC-MS^2)

Chromatographic separation was performed at a flow rate of 10 µl/min with a Luna C18 (2) reversed phase column (0.3 mm ID x 150 mm, Phenomenex Torrance, California, USA). For the elution of the lipids the gradient was set to 0 min: 0% (B); 5 min: 0% (B); 10 min: 100% (B); 30 min: 100%; 35 min: 0% (B); 70 min: 0%. The composition of solvent A was: H2O, ACN and 1 M ammonium acetate (59:40:1, v/v/v) and the pH was set to 5.6 using acetic acid (HAc). The composition of solvent B was ACN, methyl tert-butyl ether (MTBE), 2-propanol and HAc (50:40:10:0.1, v/v/v/v). Data acquisition was performed in negative ion mode using a spray voltage of 2.5 kV, a capillary temperature of 200°C, sheath gas pressure of 4 au and the S-lens radiofrequency level of 100. Precursor isolation was performed with 1 Da selection window and the resolution was set to 70.000 (FWHM at m/z 200). The MS2 spectra were recorded using adjusted collision energies for each precursor ion group. MS data interpretation (peak detection and integration) was performed with Xcalibur software (Thermo Fisher Scientific, Bremen, Germany). Transitions for each LM for the parallel reaction monitoring method are summarized in Supplemental Table S1. LM identifications were verified with the earlier reported scoring algorithm (82).

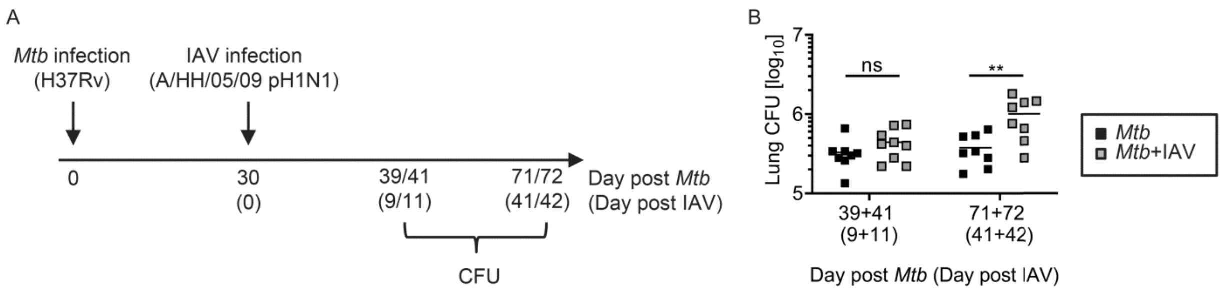

Figure S1. IAV coinfection 30 days after *Mtb* infection impairs bacterial control in the lung. (A) Schematic time-line of experimental set-up. (B) C57BL/6 mice were infected via aerosol with a low dose of *Mtb* H37Rv and 30 days later coinfected i.n. with 10^4 PFU IAV (A/HH/05/09 H1N1). At indicated time points after *Mtb* infection, lung bacterial burden was determined (n= 8-9 per group, data pooled from two independent experiments). Each data point represents one mouse. **p ≤ 0.01 determined by unpaired t-test.

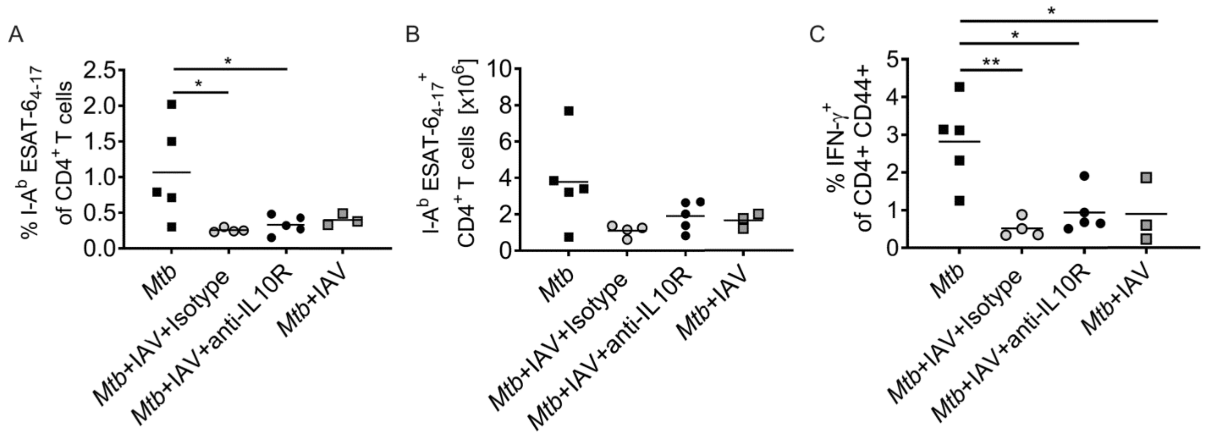

Figure S2. Anti-IL10R treatment does not restore *Mtb*-specific T cell responses. C57BL/6 mice were infected via aerosol with a low dose of *Mtb* H37Rv and 12 days later coinfected with 10^4 PFU IAV (A/HH/05/09 H1N1). 200 µg anti-IL10R antibody (1B1.3) or isotype control (polyclonal rat serum IgG) were administered i.p. on day 5 after IAV infection (day 17 *Mtb*). Lungs were analyzed by flow cytometry on day 21 *Mtb* for (A, B) I-A^b ESAT-6$_{4\text{-}17}$-specifc CD4$^+$ T cells (n= 3-5 per group, one experiment), and (C) IFN-γ producing CD4$^+$ T cells upon ex vivo stimulation with ESAT-6$_{1\text{-}20}$ peptide (n= 3-5 per group, one experiment). Each data point represents one mouse. *p ≤0.05; **p ≤ 0.01, determined by one-way ANOVA followed by Tukey's multiple-comparison test.

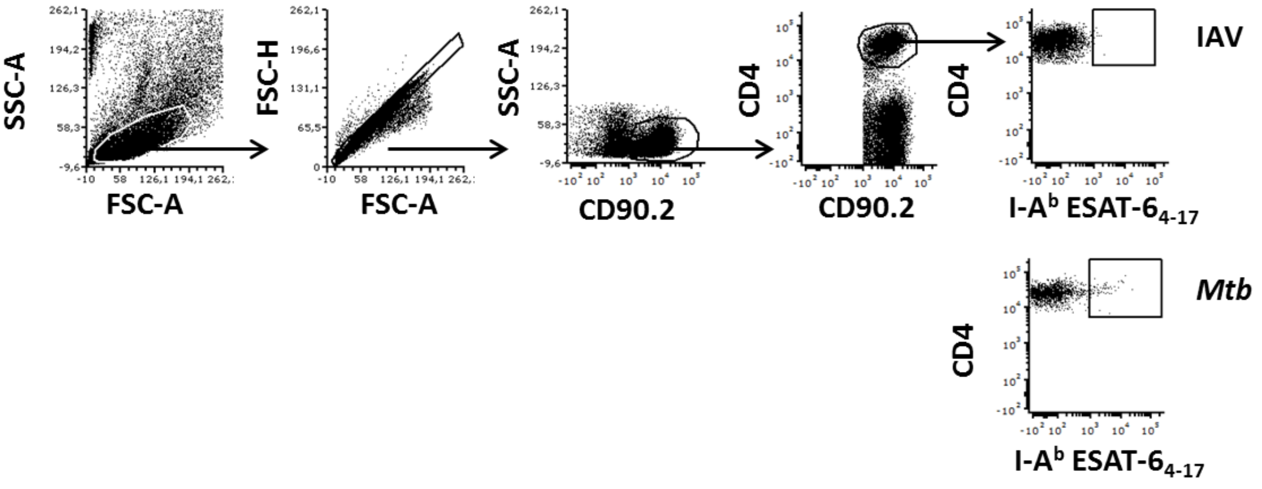

Figure S3. Flow cytometry gating strategy for determination of I-A^{b} ESAT-6$_{4-17}^{+}$ T cells. Representative flow cytometry blots for gating of I-A^{b} ESAT-6$_{4-17}^{+}$ CD4^{+} T cells isolated from lungs and dLNs. Gating strategy depicted by an example of cells isolated from the lung of an IAV-infected mouse and *Mtb*-infected mouse on day 21 after *Mtb* infection (day 9 IAV infection).

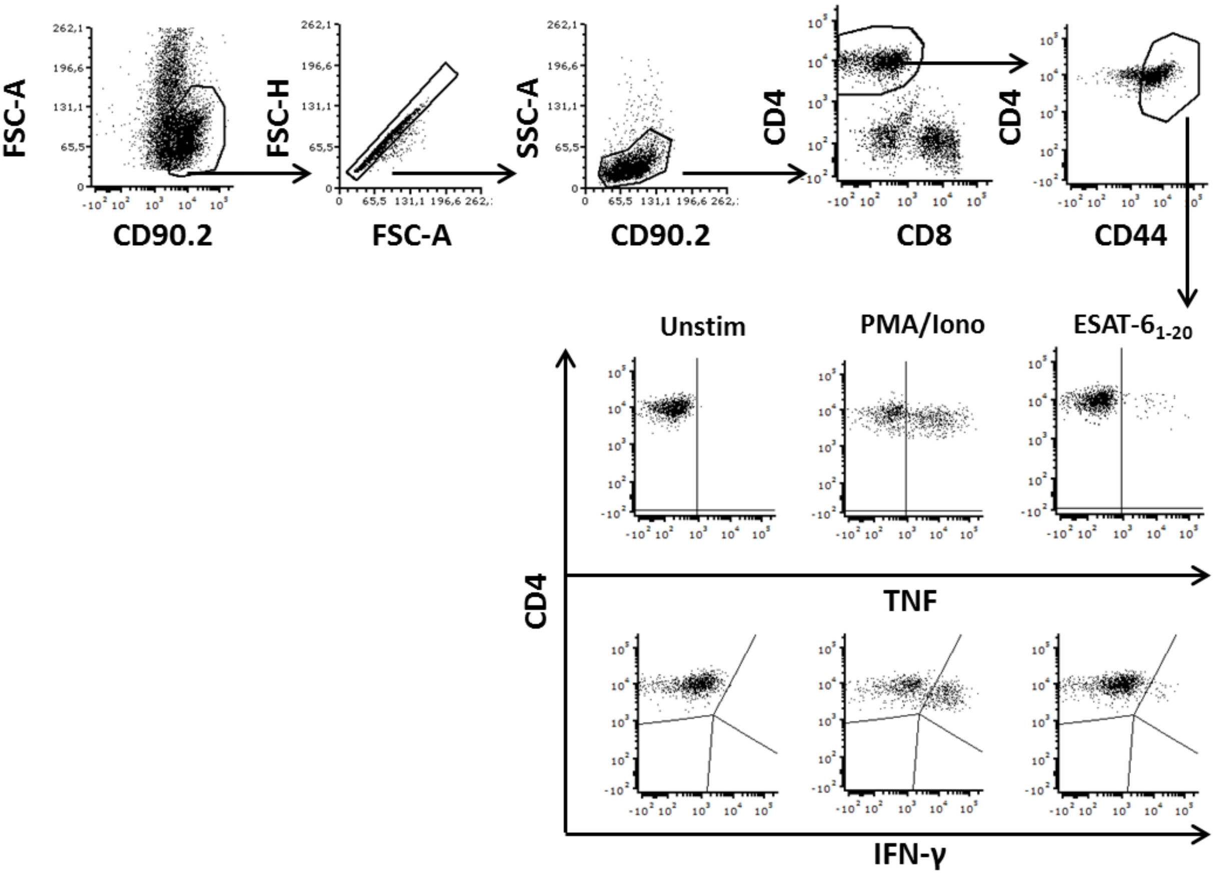

Figure S4. Flow cytometry gating strategy for determination of TNF$^+$ and IFN-γ$^+$ CD4$^+$ T cells. Representative flow cytometry blots for gating of TNF and IFN-γ$^+$ CD4$^+$ T cells isolated from lungs and dLNs. Cells from lungs of *Mtb*-infected mice on day 21 after *Mtb* infection are representatively shown.

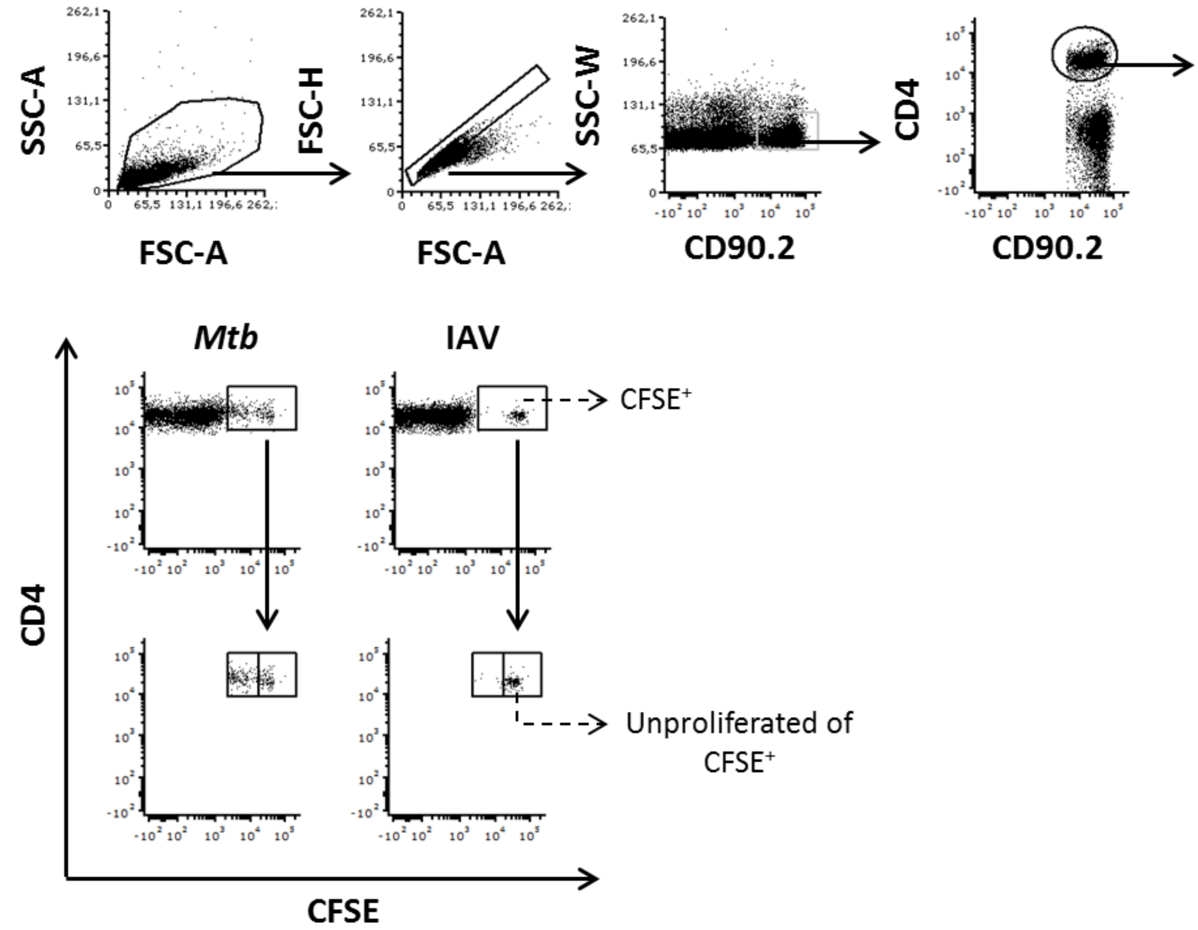

Figure S5. Flow cytometry gating strategy for determination of proliferated CD4$^+$ T cells in dLNs. Representative flow cytometry blots for detection of proliferated CFSE-labeled p25TCRtg-CD4$^+$ T cells isolated from dLNs. Proportion of proliferated cells was determined as difference of % total CFSE$^+$ - % unproliferated cells of CFSE$^+$ cells. Gating strategy depicted by an example of cells isolated from the dLN of an *Mtb*-infected mouse and IAV-infected mouse (negative control) shown for day 21 after *Mtb* infection (day 9 IAV infection).

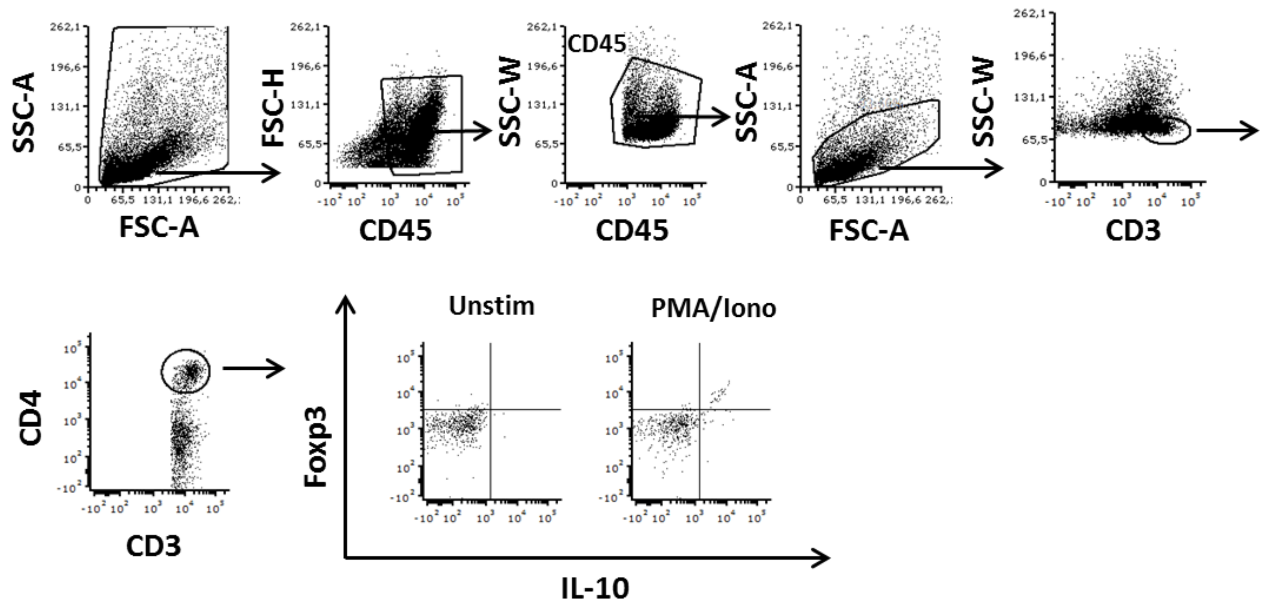

Figure S6. Flow cytometry gating strategy for determination of Foxp3$^+$ and Foxp3$^+$ IL-10$^+$ CD4$^+$ T cells. Representative flow cytometry blots for gating of Foxp3$^+$ and Foxp3$^+$ IL-10$^+$ CD4$^+$ T cells isolated from lungs. Plots are from lung cells of an *Mtb*-IAV coinfected mouse on day 18 after *Mtb* infection.

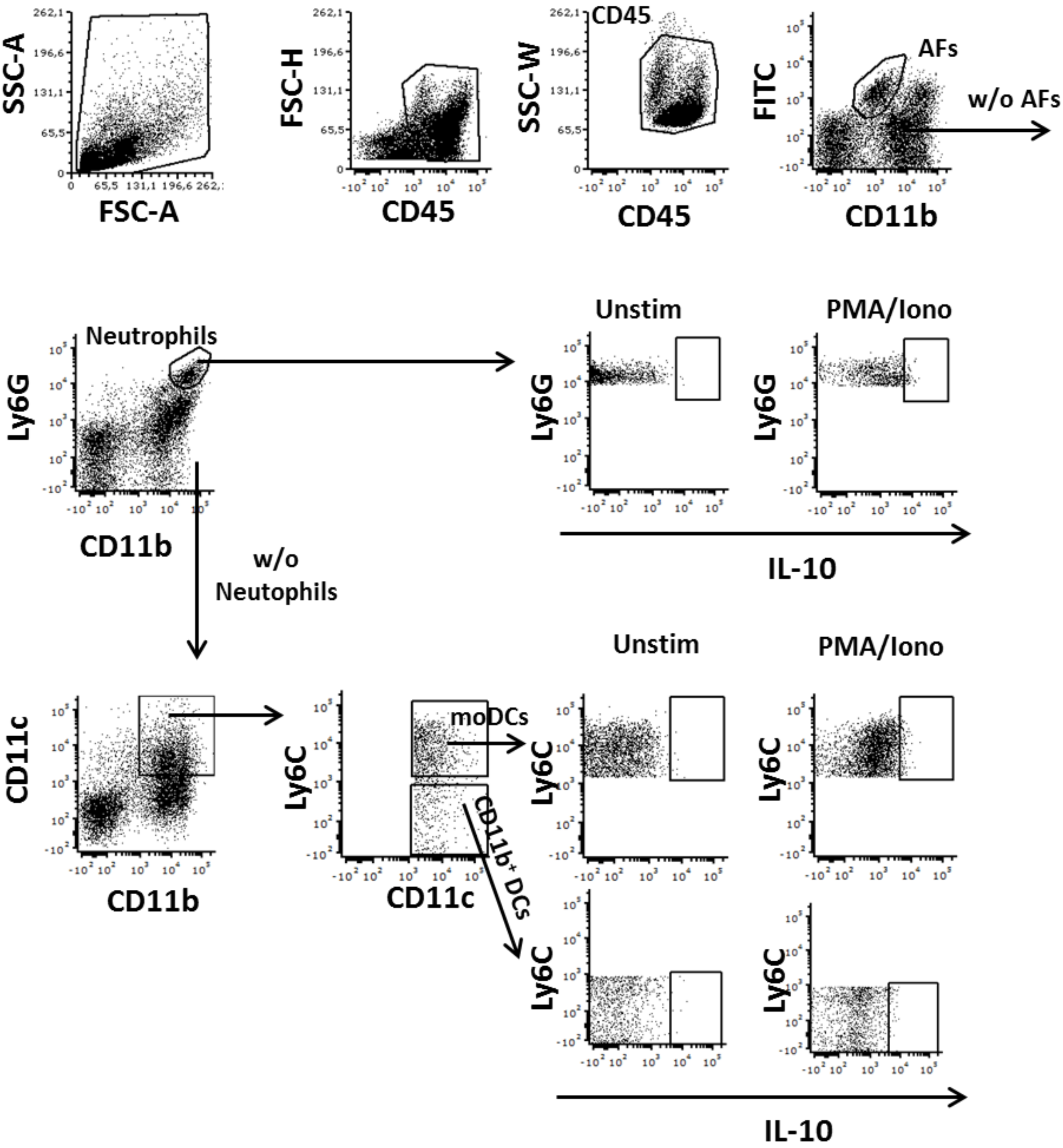

Figure S7. Flow cytometry gating strategy for determination of IL-10 producers among myeloid cells. Representative flow cytometry blots for gating of IL-10$^+$ cells among neutrophils, monocyte-derived dendritic cells (moDCs) and CD11b$^+$ DCs. Exclusion of autofluorescent cells (AFs; w/o AFs) and neutrophils (w/o neutrophils) along the gating. Plots are from lung cells of an *Mtb*-IAV coinfected mouse on day 18 after *Mtb* infection.

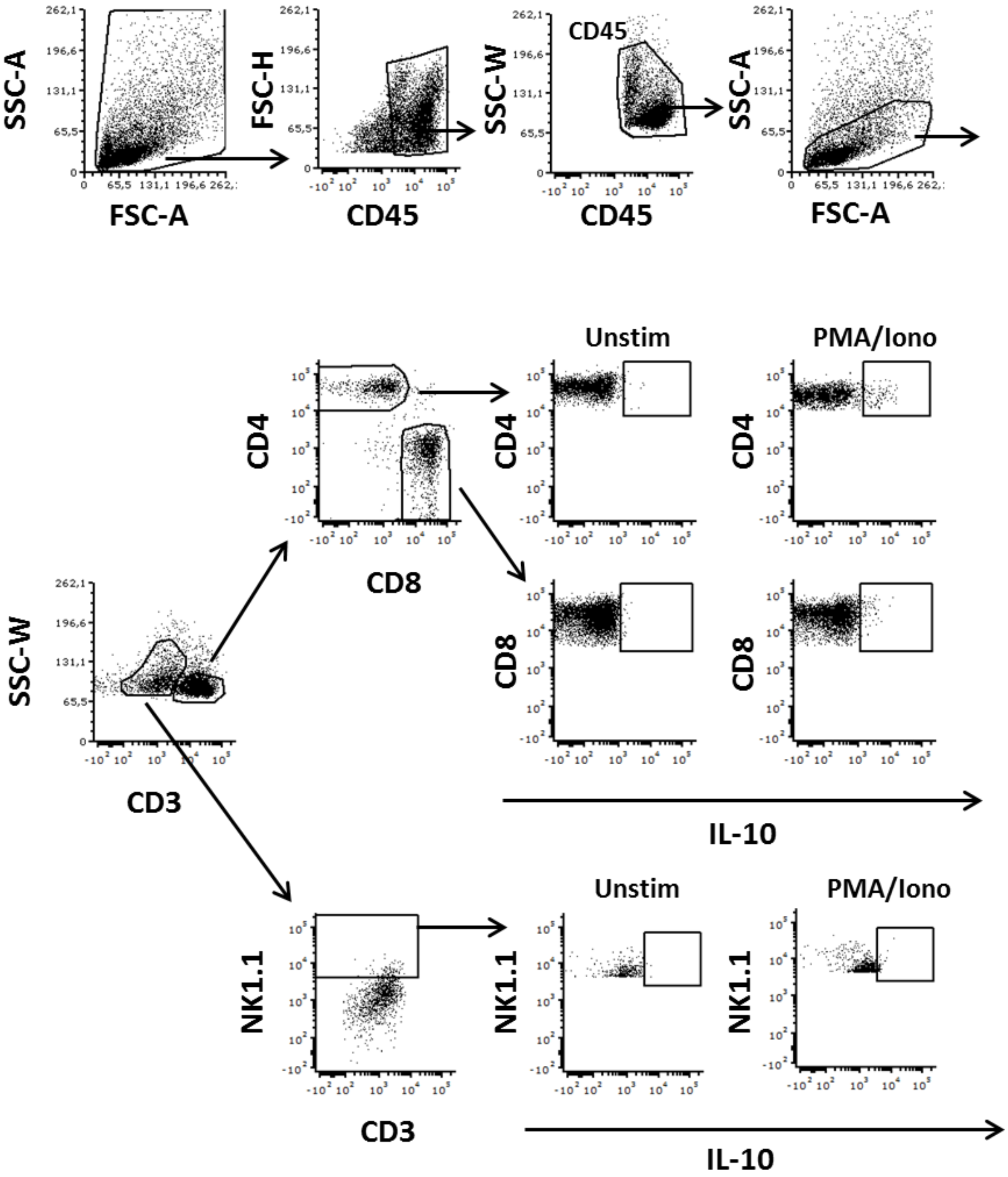

Figure S8. Flow cytometry gating strategy for determination of IL-10 producers among lymphoid cells. Representative flow cytometry blots for gating of IL-10$^+$ cells among NK cells, CD4$^+$ and CD8$^+$ T cells. Plots are from lung cells of an *Mtb*-IAV coinfected mouse on day 18 after *Mtb* infection.

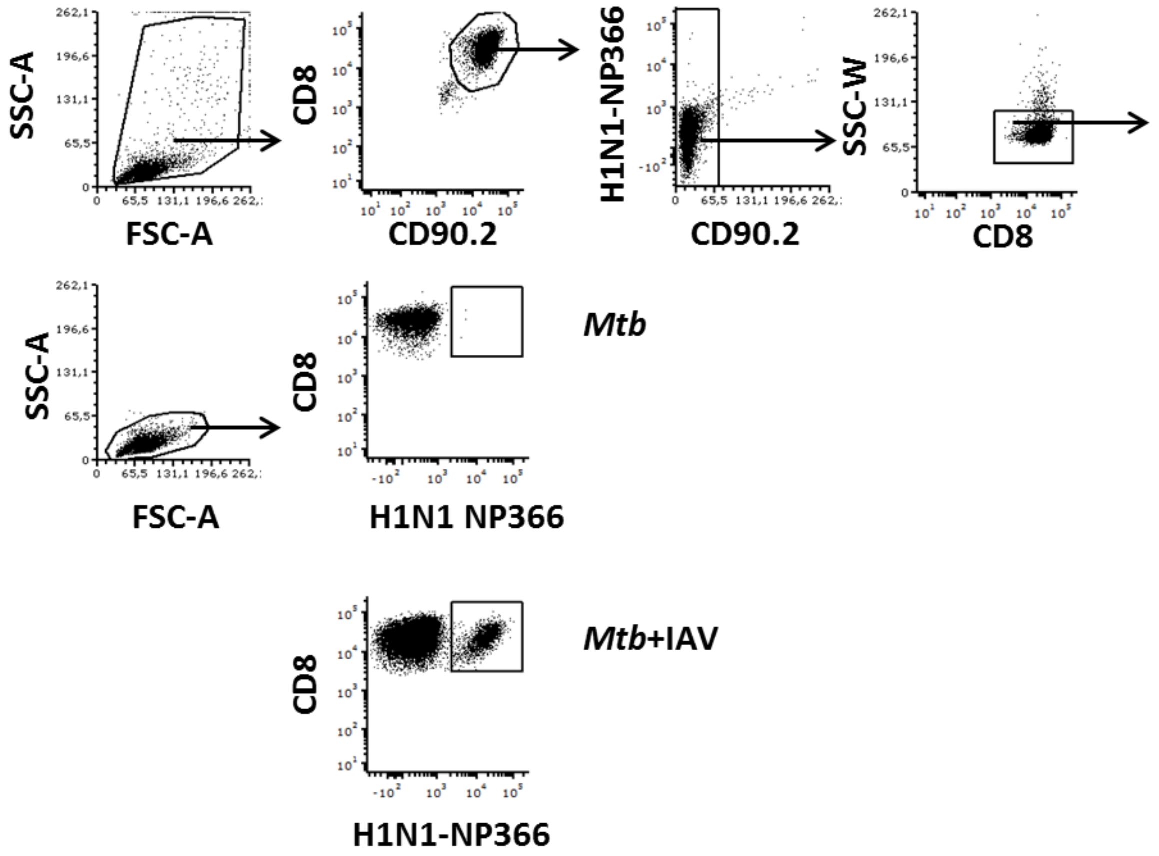

Figure S9. Flow cytometry gating strategy for determination of H1N1-NP366$^+$ (dextramer$^+$) CD8$^+$ T cells. Representative flow cytometry blots for gating of H1N1-NP366 dextramer$^+$ CD8$^+$ T cells. Plots are from lung cells of an *Mtb*- and an *Mtb*-IAV coinfected mouse on day 21 after *Mtb* infection (9 days after IAV infection).

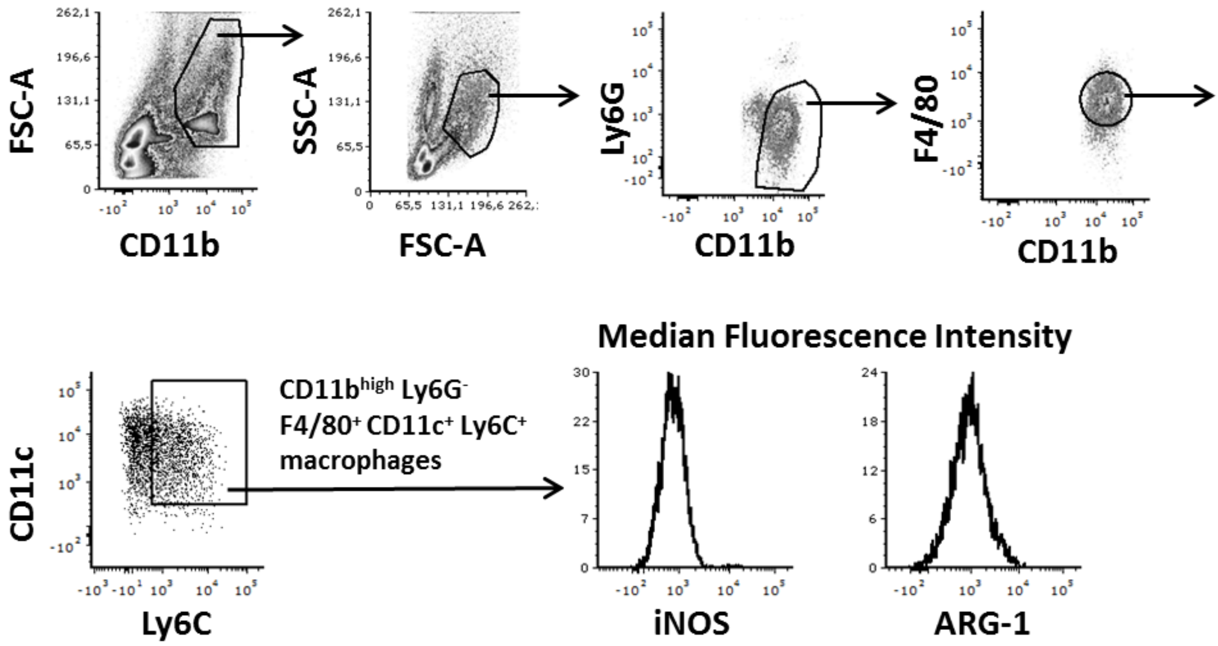

Figure S10. Flow cytometry gating strategy for determination of iNOS and ARG-1-producing macrophages. Representative flow cytometry blots for gating of macrophages and determination of median fluorescence intensity (MFI) for iNOS and ARG-1 in macrophages. Plots are from lung cells of an *Mtb*-infected lung on day 21 after *Mtb* infection (9 days after IAV infection).
